# Coordination Polymers Driven by Urea-Diisophthalate Linkers: From Hydrothermal Assembly and Structural Diversity to Catalytic Applications

**DOI:** 10.1021/acs.inorgchem.5c03114

**Published:** 2025-08-26

**Authors:** Yu Chen, Xiaoxiang Fan, Hongyu Wang, Jinzhong Gu, Marina V. Kirillova, Alexander M. Kirillov

**Affiliations:** † State Key Laboratory of Natural Product Chemistry, College of Chemistry and Chemical Engineering, 12426 Lanzhou University, Lanzhou 730000, People’s Republic of China; ‡ Nuclear Power Institute of China, Chengdu 610041, People’s Republic of China; § MINDlab: Molecular Design & Innovation Laboratory, Centro de Química Estrutural, Institute of Molecular Sciences, Departamento de Engenharia Química, Instituto Superior Técnico, 72971 Universidade de Lisboa, Av. Rovisco Pais, 1049-001 Lisbon, Portugal

## Abstract

Eight new metal­(II) coordination polymers (CPs) were assembled via hydrothermal methods from an unexplored urea-diisophthalate linker, 5,5′-(carbonylbis­(azanediyl))­diisophthalic acid (H_4_cada) and different N-donor auxiliary ligands (phen: 1,10-phenanthroline; bipy: 2,2′-bipyridine; H_2_biim: 2,2′-biimidazole; bpa: bis­(4-pyridyl)­amine; dpey: 1,2-di­(4-pyridyl)­ethylene; or dpea: 1,2-di­(4-pyridyl)­ethane). The obtained products were identified as [Mn­(μ_3_-H_2_cada)­(phen)­(H_2_O)]_*n*_·2*n*H_2_O (**1**), [Mn_2_(μ_4_-cada)­(phen)_3_(H_2_O)]_*n*_·2*n*H_2_O (**2**), [M_2_(μ_5_-cada)­(bipy)_2_(H_2_O)_2_]_*n*_·3*n*H_2_O (M = Mn (**3**) and Cd (**4**)), [Cd_2_(μ_5_-cada)­(H_2_biim)_3_]_*n*_·2*n*H_2_O (**5**), [H_2_bpa]_*n*_[Mn­(μ_3_-cada)­(H_2_O)_2_]_*n*_·3*n*H_2_O (**6**), [Co_2_(μ_6_-cada)­(μ-dpey)_0.5_(H_2_O)_4_]_*n*_·2*n*H_2_O (**7**), and [Mn_2_(μ_6_-cada)­(μ-dpea)_0.5_(H_2_O)_4_]_*n*_·2*n*H_2_O (**8**). These CPs were analyzed by standard methods, including powder and single-crystal X-ray diffraction, revealing structures ranging from 1D chains (**2**) and 2D layers (**1** and **3**–**6**) to 3D networks (**7** and **8**) with different topologies. All of the obtained CPs were also screened as heterogeneous catalysts for the C–C coupling reaction of pyridine-4-aldehyde (model substrate) with nitromethane. Substrate scope and the effects of reaction time, temperature, solvent type, catalyst loading, and recycling were explored, allowing us to identify highly efficient (up to 99% product yields), selective, and recyclable catalytic systems. This work widens the family of functional coordination polymers driven by urea-derived linkers and highlights the promising application of these materials in catalysis.

## Graphical abstract

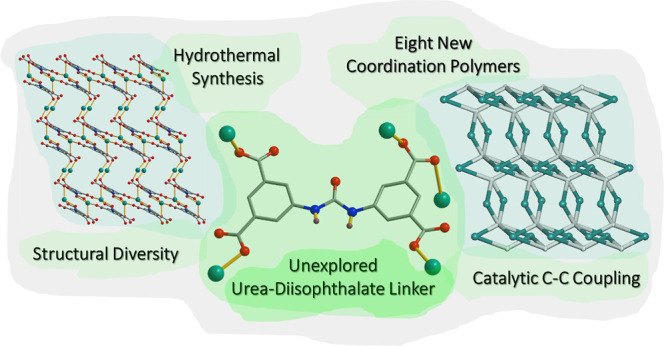

## Introduction

The rapid progress of research on coordination polymers (CPs) is attributed to their construction from versatile organic linkers and metal nodes, enabling tailored assembly and the formation of a high diversity of structural types.^1–5^ The molecular design of functional CPs has emerged as a hot topic in inorganic and coordination chemistry, driven by extensive applications of these compounds and derived materials in many areas such as, for example, gas storage and separation,^6–13^ catalysis,^14–25^ luminescent sensing,^26–29^ magnetism,^30–32^ and drug delivery and bioimaging.^33–35^ On account of facile synthesis, tunable structures, crystallinity, and chemical stability, the applications of coordination polymers have also emerged as a key area in materials science.

In particular, coordination polymers have shown growing importance in heterogeneous catalysis because of their notable selectivity, multiple sites, high activity, and ease of recovery.^14–25,36–38^ The Henry reaction, also known as the nitroaldol reaction, is a common method in organic synthesis for C–C bond formation. This reaction involves the combination of nitroalkanes with carbonyl compounds, such as aldehydes and ketones, to yield beta-nitro alcohols. Catalysts commonly used in these reactions include amines, alkoxides, or alkali metal hydroxides.^39–42^ These are not recoverable in many cases, and hence, there is a trend for developing efficient heterogeneous catalytic systems that can be used several times.

In continuation of our research line on the hydrothermal synthesis of functional CPs from commercially available polycarboxylic acids as linkers,^36,37^ in the present work, we focused our attention on urea-diisophthalic acid, 5,5′-(carbonylbis­(azanediyl))­diisophthalic acid (H_4_cada, Scheme 1), for developing a new series of coordination polymers. Apart from containing a urea-based core, H_4_cada offers eight carboxylate O-donor sites with a variety of coordination possibilities. H_4_cada also remains underexplored in the design of coordination polymers.^43,44^ Hence, in this study, we screened a number of hydrothermal reactions, resulting in the generation of eight new products: [Mn­(μ_3_-H_2_cada)­(phen)­(H_2_O)]_*n*_·2*n*H_2_O (**1**), [Mn_2_(μ_4_-cada)­(phen)_3_(H_2_O)]_*n*_·2*n*H_2_O (**2**), [M_2_(μ_5_-cada)­(bipy)_2_(H_2_O)_2_]_*n*_·3*n*H_2_O (M = Mn (**3**) and Cd (**4**)), [Cd_2_(μ_5_-cada)­(H_2_biim)_3_]_*n*_·2*n*H_2_O (**5**), [H_2_bpa]_*n*_[Mn­(μ_3_-cada)­(H_2_O)_2_]_*n*_·3*n*H_2_O (**6**), [Co_2_(μ_6_-cada)­(μ-dpey)_0.5_(H_2_O)_4_]_*n*_·2*n*H_2_O (**7**), and [Mn_2_(μ_6_-cada)­(μ-dpea)_0.5_(H_2_O)_4_]_*n*_·2*n*H_2_O (**8**). These CPs were analyzed by standard methods and evaluated as heterogeneous catalysts for the C–C coupling reaction of pyridine-4-aldehyde (the model substrate) with nitromethane. This work broadens the family of functional CPs driven by urea-derived linkers and highlights the promising application of these materials in catalysis.

**Scheme 1:**
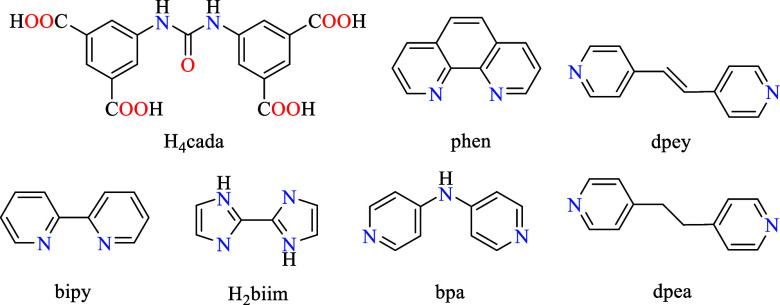
Structures of Urea-Diisophthalic Acid (H_4_cada) and Auxiliary N-Donor Ligands

## Experimental Section

### Hydrothermal Synthesis of **1**–**8**

All reagents were used as received from commercial suppliers. In a typical synthetic attempt (mmol scale; Table 1), metal­(II) chloride precursor, H_4_cada, the N-donor auxiliary ligand (phen, bipy, H_2_biim, bpa, dpey, or dpea), and sodium hydroxide were combined in deionized water and transferred to a Teflon-lined stainless steel autoclave. This was heated at 160 °C for 3 days and then cooled to room temperature at a 10 °C·h^–1^ rate. After opening the autoclave, the obtained crystals of CPs were isolated manually, washed with distilled water, and dried in air. The detailed synthetic procedures and analytical data for compounds **1**–**8** are given in the Supporting Information.

**Table 1:** Product Formulas and Compositions of the Reaction Mixtures in the Hydrothermal Synthesis of **1**–**8** ^a^

molecular formulas of CPs	metal(II) salt	auxiliary ligand (AL)	molar ratio M^2+^/H_4_edda/AL/NaOH
[Mn(μ_3_-H_2_cada)(phen)(H_2_O)]_*n*_·2*n*H_2_O (**1**)	MnCl_2_·4H_2_O	phen	1/1/1/2
[Mn_2_(μ_4_-cada)(phen)_3_(H_2_O)]_*n*_·2*n*H_2_O (**2**)	MnCl_2_·4H_2_O	phen	2/1/2/4
[Mn_2_(μ_5_-cada)(bipy)_2_(H_2_O)_2_]_*n*_·3*n*H_2_O (**3**)	MnCl_2_·4H_2_O	bipy	2/1/2/4
[Cd_2_(μ_5_-cada)(bipy)_2_(H_2_O)_2_]_*n*_·3*n*H_2_O (**4**)	CdCl_2_·H_2_O	bipy	2/1/2/4
[Cd_2_(μ_5_-cada)(H_2_biim)_3_]_*n*_·2*n*H_2_O (**5**)	CdCl_2_·H_2_O	H_2_biim	2/1/2/4
[H_2_bpa]_*n*_[Mn(μ_3_-cada)(H_2_O)_2_]_*n*_·3*n*H_2_O (**6**)	MnCl_2_·4H_2_O	bpa	2/1/2/4
[Co_2_(μ_6_-cada)(μ-dpey)_0.5_(H_2_O)_4_]_*n*_·2*n*H_2_O (**7**)	CoCl_2_·6H_2_O	dpey	2/1/2/4
[Mn_2_(μ_6_-cada)(μ-dpea)_0.5_(H_2_O)_4_]_*n*_·2*n*H_2_O (**8**)	MnCl_2_·4H_2_O	dpea	2/1/2/4

a Syntheses were performed in stainless steel (Teflon-lined, 25 mL volume) reactors, in H_2_O (10 mL), for 3 days at 160 °C.

### X-ray Crystallography

A Bruker APEX-II CCD diffractometer with graphite-monochromated MoK_α_ radiation (λ = 0.71073 Å) was used to obtain the crystallographic data for **1**–**8**. The structures were determined by direct approaches and refined using full-matrix least-squares on *F* ^2^ with the SHELXS-97 and SHELXL-97 software.^45^ C, O, and N atoms were refined anisotropically using full-matrix least-squares on *F* ^2^, while H atoms were placed in calculated positions. Crystallographic data are given in Table 2, while key bonding parameters are given in Tables S1 and S2. CCDC 2468942–2468949. Topological analysis of crystal structures was performed with ToposPro, wherein underlying nets were generated by reducing all bridging ligands to the respective centroids.^46,47^

**Table 2:** Summary of Structural Data for **1**–**8**

compound	1	2	3	4
chemical formula	C_29_H_24_MnN_4_O_12_	C_53_H_38_Mn_2_N_8_O_12_	C_37_H_34_Mn_2_N_6_O_14_	C_37_H_34_Cd_2_N_6_O_14_
formula weight	675.46	1088.78	896.55	1011.47
crystal system	monoclinic	triclinic	monoclinic	monoclinic
space group	*P*2_1_/*c*	*P*–1	*P*2/*c*	*P*2/*c*
*a*/Å	7.4587(2)	10.74070(10)	15.9315(4)	16.0679(4)
*b*/Å	21.6169(4)	15.1929(2)	11.9560(3)	11.9678(3)
*c*/Å	16.5164(4)	16.8058(2)	20.3365(5)	20.4533(15)
α/°	90	115.8010(10)	90	90
β/°	92.042(2)	99.1480(10)	99.632(3)	98.544(4)
γ/°	90	98.0840(10)	90	90
*V*/Å^3^	2661.31(11)	2368.73(5)	3819.03(17)	3889.5(3)
*T*/K	294(2)	302(2)	302(2)	279(2)
Z	4	2	4	4
*D*c/g cm^–3^	1.686	1.501	1.497	1.666
μ/mm^–1^	4.744	4.958	0.732	1.163
*F*(000)	1388	1096	1760	1944
refl. measured	4760	8593	7502	7244
unique refl. (*R*_int_)	3611 (0.0540)	7685 (0.0337)	5974 (0.0340)	5907 (0.0333)
GOF on *F* ^2^	1.028	1.065	1.077	1.076
*R*_1_[*I* > 2σ(*I*)]^*a*^	0.0495	0.0396	0.0410	0.0271
w*R*_2_[*I* > 2σ(*I*)]^*b*^	0.1217	0.1145	0.0524	0.0692
				

compound	**5**	**6**	**7**	**8**
chemical formula	C_35_H_30_Cd_2_N_14_O_11_	C_27_H_29_MnN_5_O_14_	C_23_H_25_Co_2_N_3_O_15_	C_46_H_52_Mn_4_N_6_O_30_
formula weight	1047.50	702.49	701.29	1388.69
crystal system	triclinic	monoclinic	triclinic	triclinic
space group	*P*–1	*P*2_1_/*n*	*P*–1	*P*–1
*a*/Å	7.6383(2)	7.7293(3)	10.4636(2)	10.6992(2)
*b*/Å	13.3424(4)	14.6568(5)	10.8720(2)	10.98890(10)
*c*/Å	20.1486(6)	25.6969(11)	11.9856(2)	12.14660(10)
α/°	95.696(2)	90	86.3780(10)	87.2260(10)
β/°	95.433(2)	96.187(4)	71.326(2)	70.6170(10)
γ/°	105.154(3)	90	87.175(2)	87.9420(10)
*V*/Å^3^	1956.59(11)	2894.2(2)	1288.52(4)	1345.29(3)
*T*/K	302(2)	303(2)	292(2)	303(2)
Z	2	4	2	1
*D*c/g cm^–3^	1.717	1.612	1.715	1.714
μ/mm^–1^	1.160	0.539	10.765	8.379
*F*(000)	1004	1452	676	710
refl. measured	7274	5381	4723	4889
unique refl. (*R*_int_)	5694 (0.0376)	4256 (0.0417)	4006 (0.0370)	4529 (0.0300)
GOF on *F* ^2^	1.036	1.033	1.036	1.050
*R*_1_ [*I* > 2σ(*I*)]^*a*^	0.0386	0.0356	0.0345	0.0381
*wR*_2_ [*I* > 2σ(*I*)]^*b*^	0.0966	0.0816	0.0799	0.1124

### Catalytic Studies

In a typical catalytic test for the Henry reaction, pyridine-4-aldehyde (0.50 mmol; model substrate), nitromethane (2.0 mmol), and coordination polymer catalyst (typically 4.0 mol %) were combined in methanol (1.0 mL) in a flask equipped with a reflux condenser. The obtained suspension was stirred at 70 °C for 1–12 h. After the reaction was completed, the catalyst was isolated by centrifugation. The reaction solution was evaporated in vacuo to give a crude solid. This was dissolved in CDCl_3_ and analyzed by ^1^H NMR spectroscopy using the JNM ECS 400 M spectrometer. Further details on product analysis and quantification are given in Figure S3. In the experiments on catalyst recycling, the catalyst was separated by centrifugation, washed with methanol, dried at 25 °C in air, and then reused in subsequent tests. Blank tests and substrate scope studies with other aldehydes were also performed following the above-described procedure.

## Results and Discussion

### Hydrothermal Synthesis of **1**–**8**

To explore 5,5′-(carbonylbis­(azanediyl))­diisophthalic acid (H_4_cada) as a versatile but still little investigated urea-diisophthalic acid linker in the molecular design of coordination polymers, we probed a number of reactions under hydrothermal conditions. In these reactions, we used different combinations of a metal­(II) chloride precursor (MnCl_2_·4H_2_O, CdCl_2_·H_2_O, or CoCl_2_·6H_2_O), H_4_cada as a primary linker, sodium hydroxide as a deprotonating agent, and N-donor auxiliary ligand (phen, bipy, H_2_biim, bpa, dpey, and dpea) acting also as a mediator of crystallization. Different molar ratios of reagents were also explored. The reaction mixtures in water were heated at 160 °C for 3 days, followed by slow cooling and crystallization of coordination polymers. The selection of Mn, Cd, and Co as metal nodes is explained by their low cost, rich coordination chemistry with carboxylate ligands, and high reactivity of their coordination polymers in different types of catalytic reactions.^20,36,38,40–42^ The use of simple and easily available N-donor auxiliary ligands (Scheme 1) is governed by their structure-stabilizing effect on the coordination polymers. Also, these N-donor ligands act as mediators of crystallization during the self-assembly synthesis.^16,31,32,36,37^ Eight synthetic attempts were successful and resulted in the isolation of CPs **1**–**8** (Table 1) as pure microcrystalline solids that were characterized by standard methods. Phase purity of compounds **1**–**8** was confirmed by PXRD analyses. The obtained diffractograms match the patterns simulated from single-crystal X-ray data (Figure S2, Supporting Information). The structural variations observed in CPs **1**–**8** evolve from different coordination modes of metal­(II) centers and the type of auxiliary ligand used. In addition, the H_2_cada^2–^/cada^4–^ linkers reveal six distinct coordination modes in compounds **1**–**8** (Scheme 2).

**Scheme 2:**
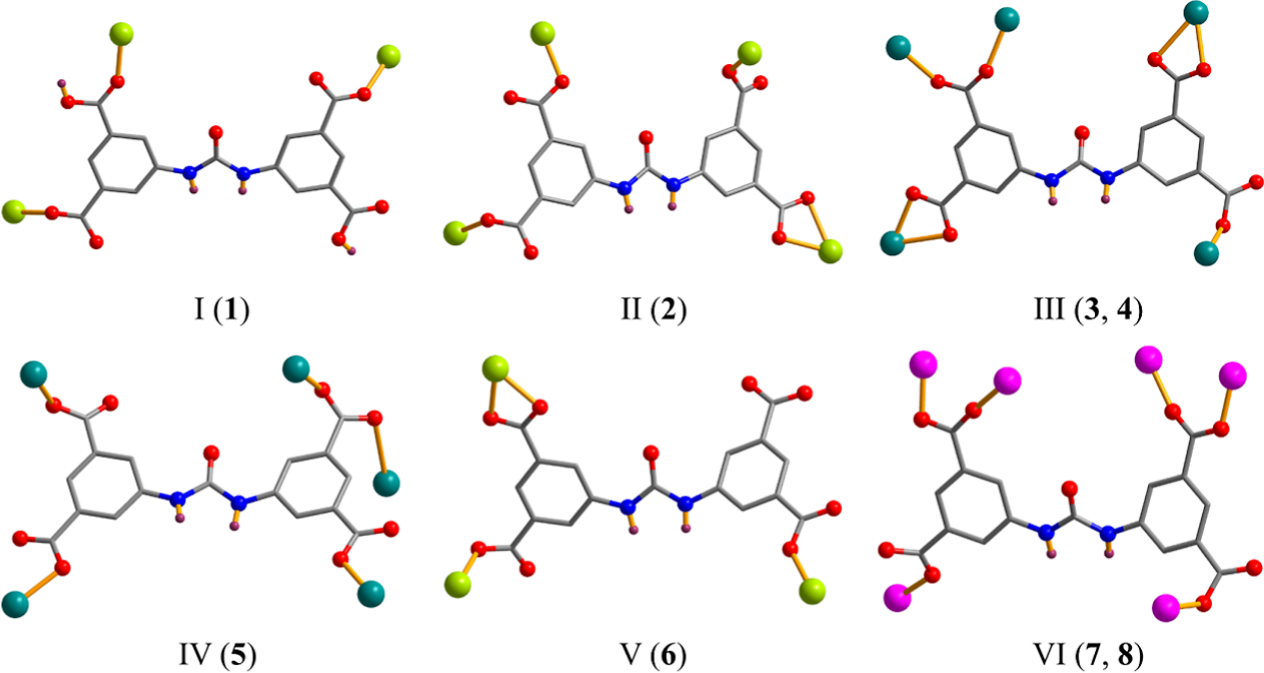
Coordination Modes of H_2_cada^2–^/cada^4–^ Linkers in **1**–**8**

### Structural Features

#### [Mn­(μ_3_-H_2_cada)­(phen)­(H_2_O)]_n_·2nH_2_O (**1**)

In this 2D CP (Figure 1), the asymmetric unit contains μ_3_-H_2_cada^2–^, phen, and H_2_O ligands per manganese­(II) center. The Mn1 atom features a distorted octahedral {MnN_2_O_4_} geometry with three carboxylate O atoms from three μ_3_-H_2_cada^2–^ units, two N_phen_ donors, and one water ligand (Figure 1a). The H_2_cada^2–^ ligand exhibits a μ_3_-coordination mode (mode I, Scheme 2) and interlinks the Mn1 centers into a 2D layer (Figure 1b). Its topological analysis reveals a uninodal 3-linked net with the **fes** topology and point symbol of (4.8^2^) (Figure 1c).

**Figure 1:**
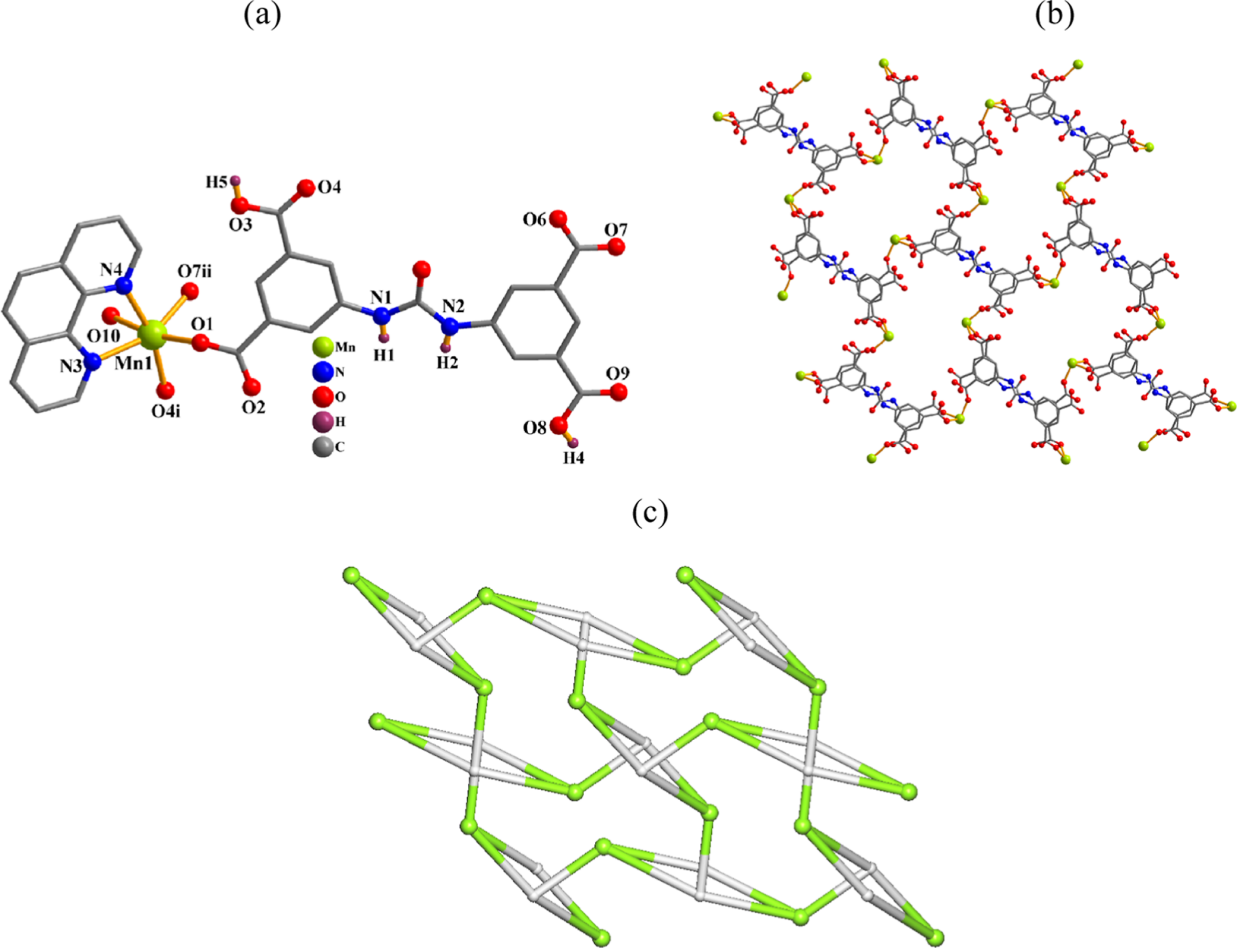
Structure of **1**. (a) Coordination environment around the Mn­(II) center. (b) 2D layer and (c) its topological graph showing the **fes** net. Details: H atoms (a,b) and phen moieties (b) are omitted; (b,c) rotated views along the *a* axis; (c) Mn (green), centroids of μ_3_-H_2_cada^2–^ linkers (gray).

#### [Mn_2_(μ_4_-cada)­(phen)_3_(H_2_O)]_n_·2nH_2_O (**2**)

The 1D coordination polymer **2** depicted in Figure 2 possesses two Mn­(II) atoms, one μ_4_-cada^2–^ block, three phen ligands, and one coordinated water molecule within the asymmetric unit. The 6-coordinated Mn1 center reveals a deformed octahedral {MnN_2_O_4_} geometry. It is filled by four O atoms from three μ_4_-cada^4–^ blocks, and two N_phen_ donors (Figure 2a). The Mn2 center is also 6-coordinated and shows a distorted octahedral {MnN_4_O_2_} environment (Figure 2a), taken by four N_phen_ atoms, one O donor from μ_4_-cada^4–^, and a terminal water ligand. The cada^4–^ linker is fully deprotonated and exhibits a μ_4_-coordination mode (type II, Scheme 2). These μ_4_-cada^4–^ blocks connect adjacent Mn atoms to form a 1D double chain structure (Figure 2b). This possesses the (4,4)­(0,2) topology with a point symbol of (4^2^.6) (Figure 2c).

**Figure 2:**
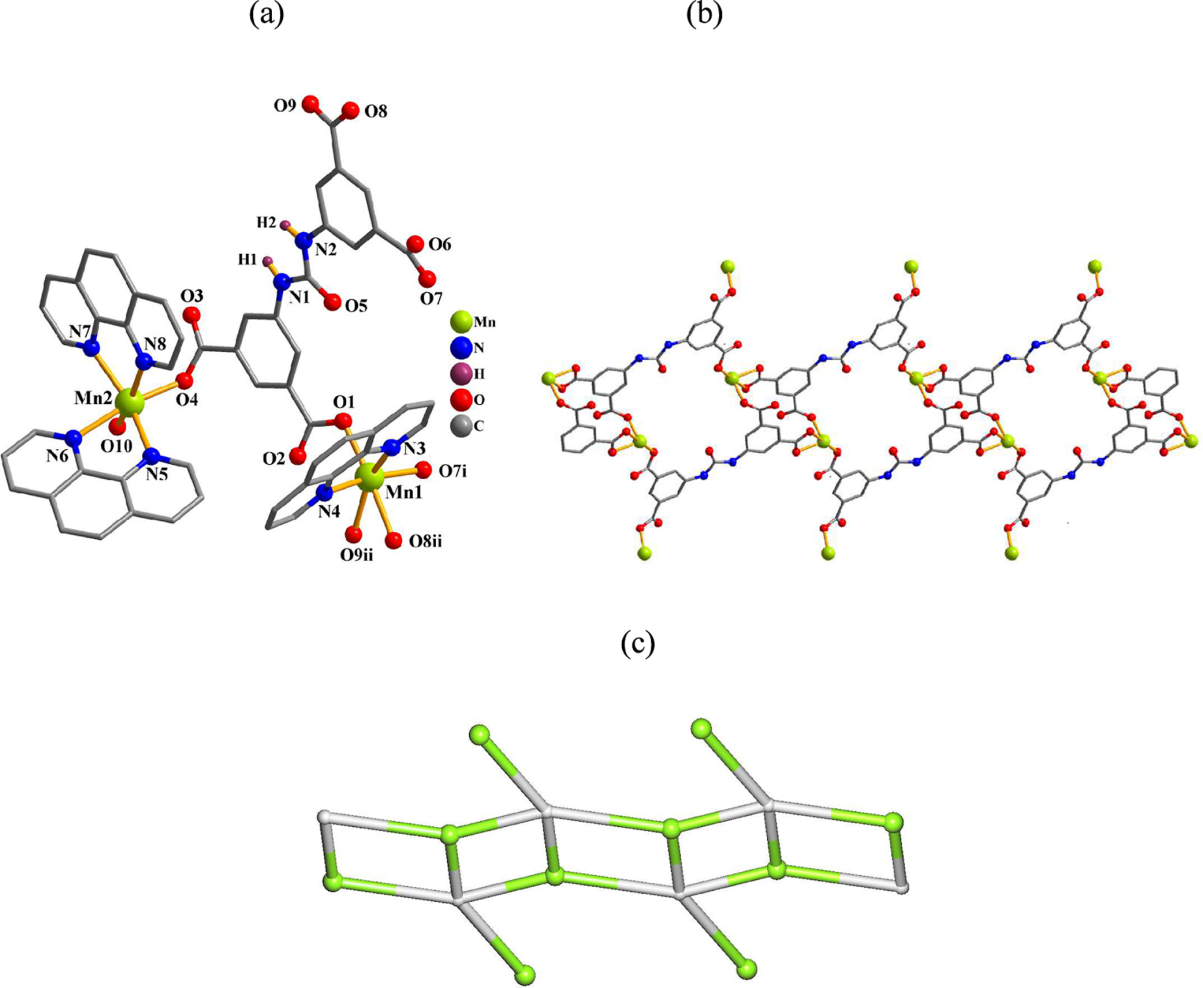
Structure of **2**. (a) Coordination environment around the Mn­(II) atoms. (b) 1D double chain and (c) its topological graph showing the (4,4)­(0,2) topology. Details: H atoms (a,b) and phen moieties (b) are omitted; (b,c) rotated views along the *c* axis; (c) Mn (green), centroids of μ_4_-cada^4–^ linkers (gray).

#### [M_2_(μ_5_-cada)­(bipy)_2_(H_2_O)_2_]_n_·3nH_2_O (M = Mn (**3**) and Cd (**4**))

These 2D coordination polymers are isostructural (Table 2) and, therefore, only the structure of **4** is discussed here (Figure 3). In the asymmetric unit of **4**, there are two Cd­(II) atoms, one μ_5_-cada^4–^ block, and two bipy and one H_2_O ligands. The Cd1 center adopts a distorted octahedral {CdN_2_O_4_} environment filled by three carboxylate O atoms from three μ_5_-cada^4–^ blocks, two N_bipy_ donors, and a H_2_O ligand (Figure 3a). The Cd2 center is seven-coordinated and displays a deformed pentagonal-bipyramidal {CdN_2_O_5_} environment, which is composed of four carboxylate O donors from two μ_5_-cada^4–^ linkers, a pair of N_bipy_ atoms, and a water ligand. In **4**, the μ_5_-cada^4–^ linkers display a coordination mode III (Scheme 2) and interconnect the Cd1 and Cd2 centers into a 2D corrugated layer (Figure 3b). From a topological perspective, it can be classified as a binodal 3,5-linked net with the 3,5L2 topology and a point symbol of (4^2^.6^7^.8)­(4^2^.6) (Figure 3c).

**Figure 3:**
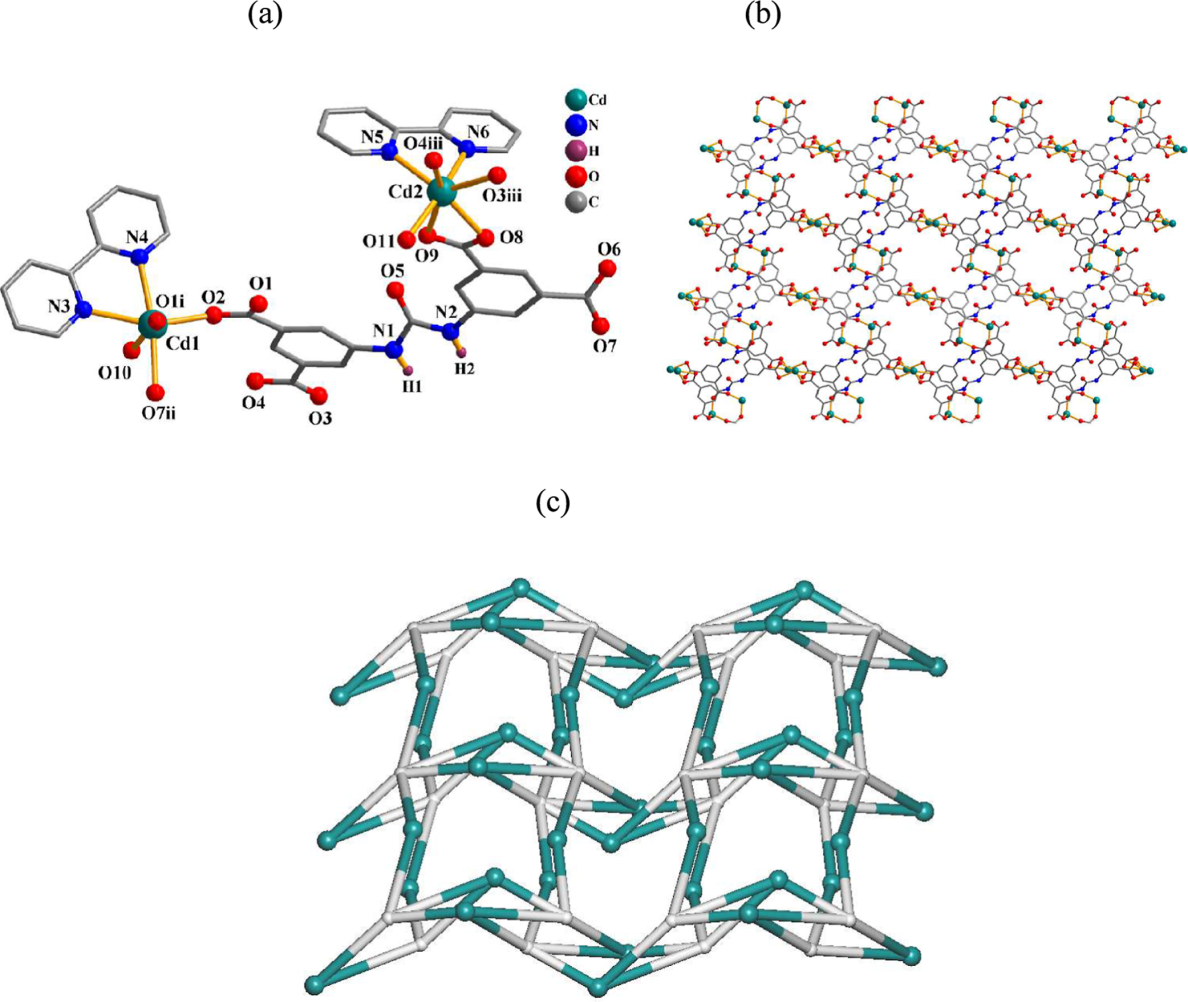
Structure of **4**. (a) Coordination environment around the Cd­(II) atoms. (b) 2D layer and (c) its topological graph showing the 3,5L2 topology. Details: H atoms (a,b) and bipy moieties (b) are omitted; (b,c) rotated views along the *b* axis; (c) Cd (turquoise), centroids of μ_5_-cada^4–^ linkers (gray).

#### [Cd_2_(μ_5_-cada)­(H_2_biim)_3_]_n_·2nH_2_O (**5**)

The structure of this 2D coordination polymer (Figure 4) comprises two Cd­(II) centers, one μ_5_-cada^4–^ block, and three H_2_biim ligands per asymmetric unit. The six-coordinated *C*_d1_ center shows a deformed octahedral {CdN_2_O_4_} environment, which is populated by four carboxylate oxygen atoms from three μ_5_-cada^4–^ linkers and two N donors from H_2_biim (Figure 4a). The Cd2 center is also six-coordinated in an octahedral {CdN_4_O_2_} geometry, with two carboxylate O atoms from two μ_5_-cada^4–^ linkers and four N donors from a pair of H_2_biim ligands (Figure 4a). The cada^4–^ blocks function as μ_5_-linkers (mode IV, Scheme 2) and assemble the Cd atoms into a complex 2D layer (Figure 4b). Its topological analysis reveals a binodal 3,4-linked layer (Figure 4c) with the 3,4L83 topology and a point symbol of (4^2^.6^3^.8)­(4^2^.6).

**Figure 4:**
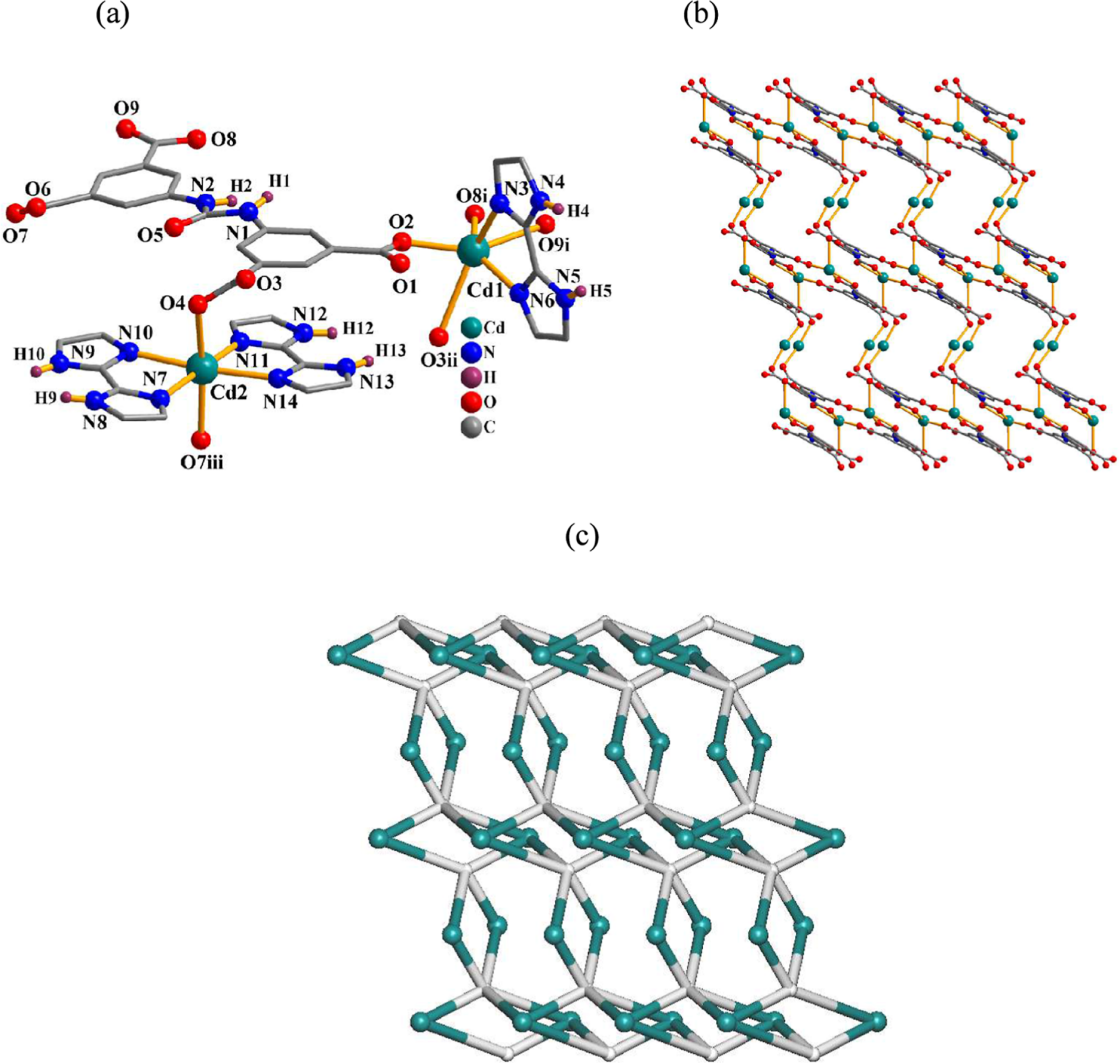
Structure of **5**. (a) Coordination environment around the Cd­(II) atoms. (b) 2D layer and (c) its topological graph showing the 3,4L83 topology. Details: H atoms (a,b) and H_2_biim moieties (b) are omitted; rotated views along the *c* axis; (c) Cd (turquoise), centroids of μ_5_-cada^4–^ linkers (gray).

#### [H_2_bpa]_n_[Mn­(μ_3_-cada)­(H_2_O)_2_]_n_·3nH_2_O (**6**)

The crystal structure of this ionic compound is composed of anionic 2D layers of [Mn­(μ_3_-cada)­(H_2_O)_2_]_*n*_^2–^ and [H_2_bpa]^2+^ cations (Figure 5). The six-coordinated Mn1 center discloses a distorted octahedral {MnO_6_} geometry, which is based on four carboxylate O atoms from three μ_3_-cada^4–^ blocks and two H_2_O ligands (Figure 5a). The cada^4–^ linkers exhibit a μ_3_-coordination fashion (mode V, Scheme 2) and assemble the Mn1 centers into a 2D network (Figure 5b). It can be defined as a mononodal net with a **fes** topology and point symbol of (4.8^2^) (Figure 5c).

**Figure 5:**
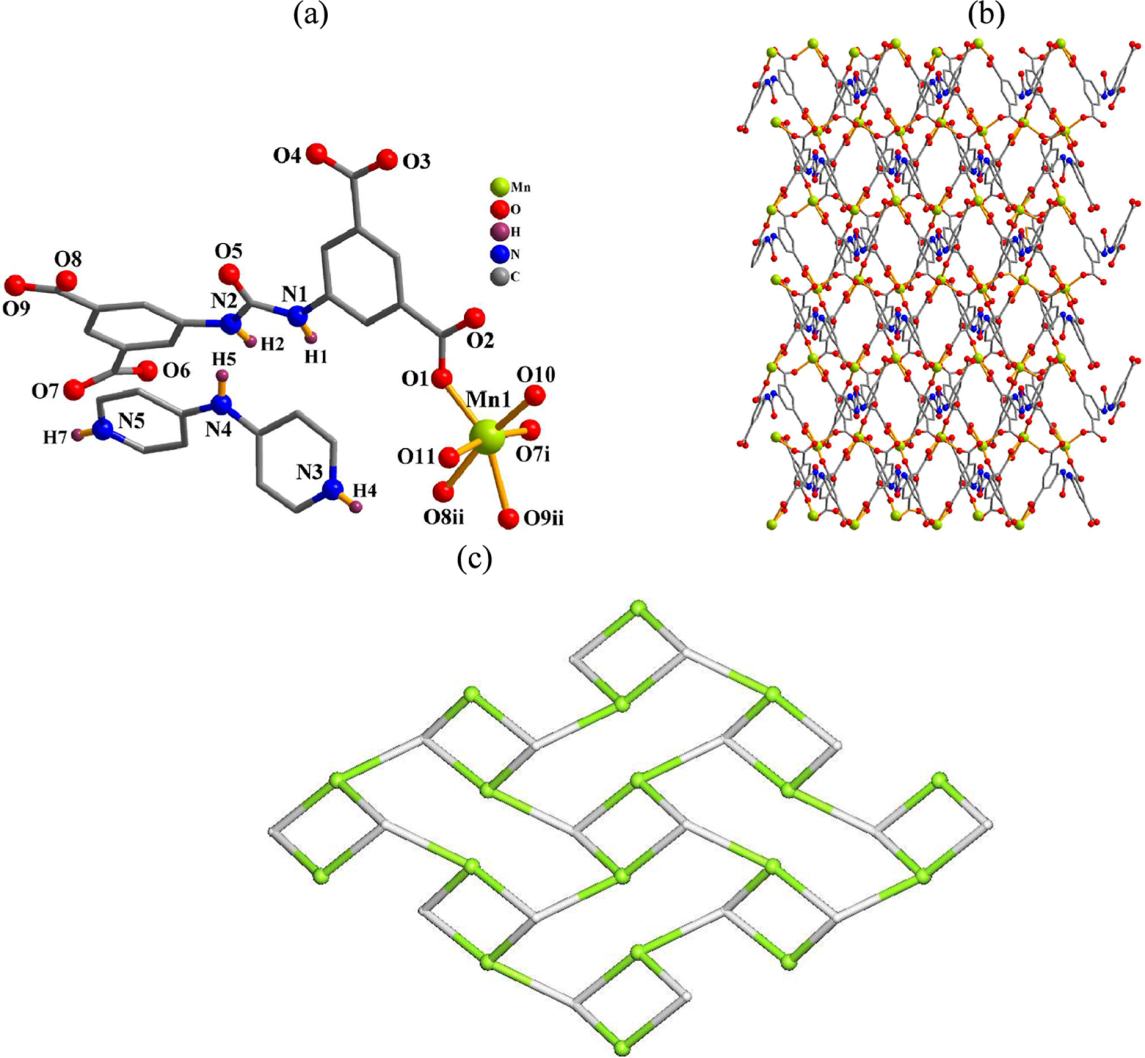
Structure of **6**. (a) Coordination environment around the Mn­(II) atom. (b) 2D layer and (c) its topological graph showing the **fes** topology. Details: H atoms (a,b) and H_2_bpa^2+^ cations (b) are omitted; views along the *c* (b) or *a* (c) axis; (c) Mn (green), centroids of μ_3_-cada^4–^ linkers (gray).

#### [Co_2_(μ_6_-cada)­(μ-dpey)_0.5_(H_2_O)_4_]_n_·2nH_2_O (**7**) and [Mn_2_(μ_6_-cada) (μ-dpea)_0.5_(H_2_O)_4_]_n_·2nH_2_O (**8**)

As these 3D coordination polymers have similar structures, only compound **7** is discussed herein (Figure 6). There are two cobalt­(II) centers, one μ_6_-cada^4–^ block, half of the μ-dpey ligand, and four coordinated water molecules in the asymmetric unit of **7**. The Co1 center is six-coordinated in a distorted octahedral {CoO_6_} fashion, involving three carboxylate O atoms from three μ_6_-cada^4–^ units and three H_2_O ligands (Figure 6a). The Co2 center is five-coordinated and has a square pyramidal {CoNO_4_} environment, which is occupied by three O atoms from three μ_6_-cada^4–^ linkers, an N_dpey_ donor, and a water ligand (Figure 6a). The cada^4–^ linkers exhibit μ_6_-coordination (mode VI, Scheme 2). A dimeric Co_2_ subunit with a Co···Co separation of 3.672(3) Å is formed by bridging the Co1 and Co2 centers through two carboxylate groups from two μ_6_-cada^4–^ ligands. These Co_2_ subunits are then interlinked by the μ_6_-cada^4–^ ligands into 2D motifs, which are further held together by the μ-dpey pillars into a 3D metal–organic network (Figure 6b). It can be classified as a trinodal 3,4,6-connected net (Figure 6c) with a unique topology and point symbol of (4^2^.6^3^.8)­(4^2^.6)­(4^8^.6^6^.8).

**Figure 6:**
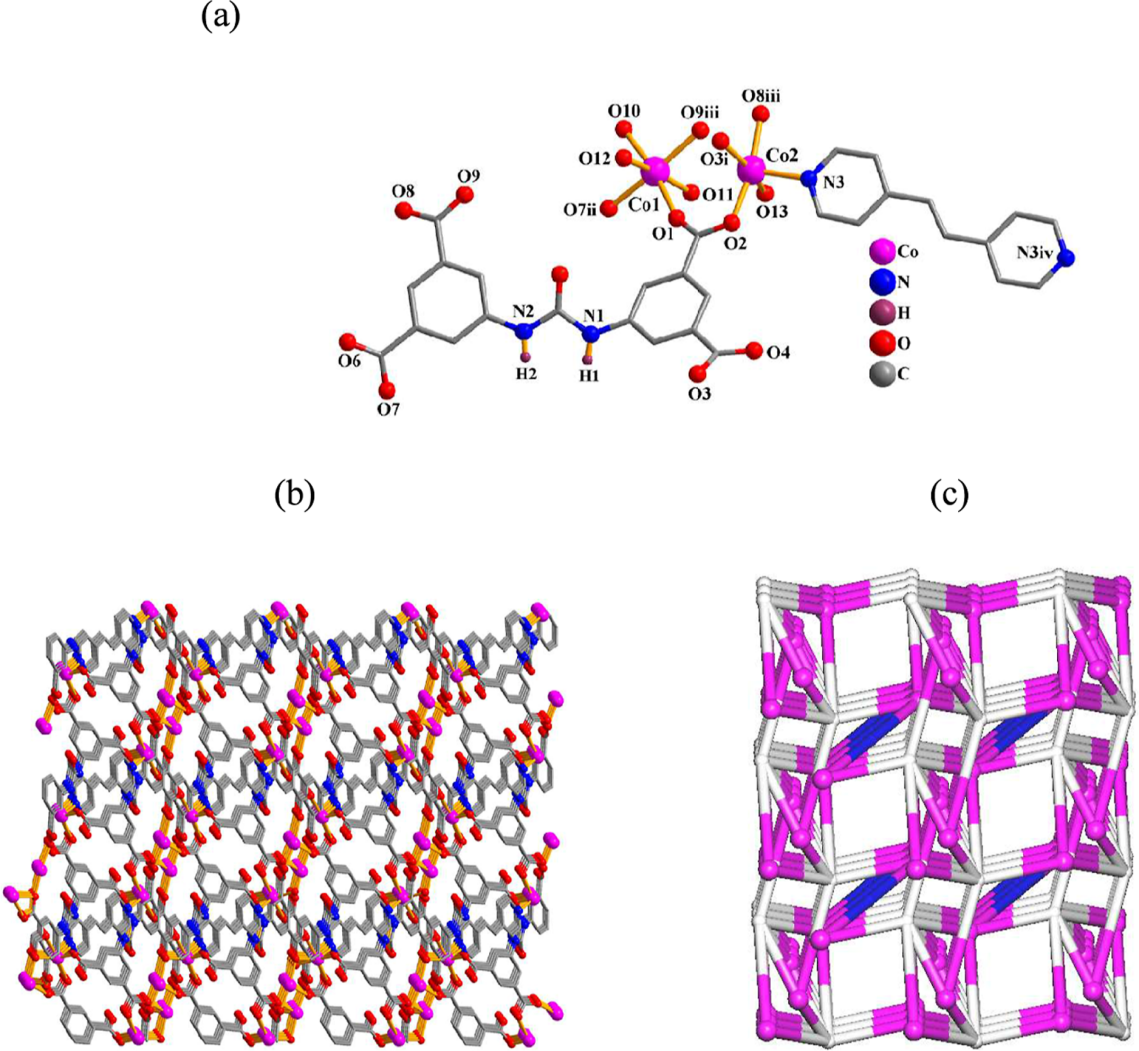
Structure of **7**. (a) Coordination environment around the Co­(II) atoms. (b) 3D network and its topological graph. Details: H atoms (a,b) are omitted; (b,c) views along the *c* axis; (c) Co (magenta), centroids of μ_6_-cada^4–^ (gray) and μ-dpey (blue) linkers.

### Thermal Analysis

Thermogravimetric analysis (TGA) of compounds **1**–**8** was carried out under an inert N_2_ atmosphere over the 25–800 °C temperature range (Figure 7). The TGA curve of **1** reveals a mass loss between 52 and 209 °C, attributable to the release of two crystallization and one coordinated H_2_O molecules (observed 7.8%; calculated 8.0%), with decomposition starting at 267 °C. Compound **2** undergoes a disintegration above 251 °C, preceded by the release of two lattice and one coordinated H_2_O molecules between 41 and 211 °C (observed 5.0%; calculated 4.9%). For CP **3**, a decrease of weight (observed 10.1%; calculated 10.0%) between 53 and 138 °C is due to the removal of three crystallization and two coordinated H_2_O molecules, followed by decomposition above 284 °C. The TGA curve of **4** reveals a mass loss corresponding to the removal of three lattice and two coordinated H_2_O molecules between 34 and 147 °C (observed 9.2%; calculated 8.9%), with the decomposition of the dehydrated solid occurring after 287 °C. Thermal analysis of **5** shows a weight loss between 45 and 214 °C, attributable to the release of two crystallization H_2_O molecules (observed 3.7%; calculated 3.4%), with the decomposition beginning at 300 °C. Coordination polymer **6** undergoes disintegration above 236 °C, preceded by a release of three lattice and two coordinated H_2_O molecules between 44 and 179 °C (observed 13.0%; calculated 12.8%). For **7**, a mass decrease (observed 15.2%; calculated 15.4%) between 33 and 164 °C is due to the removal of two crystallization and four coordinated H_2_O molecules, followed by decomposition starting at 312 °C. The TGA curve of **8** exhibits a weight loss between 40 and 131 °C, which is associated with the release of two crystallization and four coordinated H_2_O molecules (observed 15.5%; calculated 15.6%). The dehydrated metal–organic network remains stable up to 420 °C.

**Figure 7:**
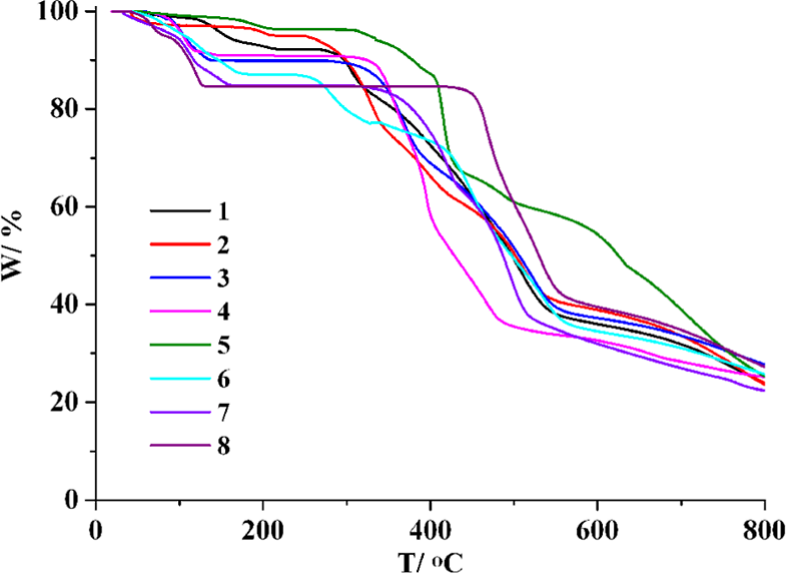
TGA curves for CPs **1**–**8**.

### Catalytic Activity in the Henry Reaction

As various coordination polymers are known to catalyze the coupling reactions between aldehydes and nitroalkanes (Henry reactions),^20,36,38,40–42,48–54^ we evaluated the potential of compounds **1**–**8** as prospective heterogeneous catalysts in this type of C–C coupling reactions (Table 3). Pyridine-4-aldehyde was selected as a model substrate, and its reaction with nitromethane was investigated at 70 °C in methanol, resulting in the formation of 2-nitro-1-(pyridin-4-yl)­ethan-1-ol as a product (Scheme 3, Table 3). In addition, for the most active catalyst, we studied the influence of different reaction parameters, including temperature, loading of the catalyst and its recyclability, reaction time, solvent choice, and substrate scope (Table 4).

**Table 3:** Coupling of Pyridine-4-aldehyde with Nitromethane Catalyzed by **1**–**8** ^a^

entry	catalyst	product yield, %^b^
1	**1**	84
2	**2**	86
3	**3**	85
4	**4**	80
5	**5**	99
6	**6**	78
7	**7**	93
8	**8**	91
9	blank	3
10	CdCl_2_	14
11	H_4_cada	17

a Conditions: pyridine-4-aldehyde (0.5 mmol), nitromethane (2.0 mmol), catalyst (4 mol %), methanol solvent (1.0 mL), 12 h, 70 °C.

b Yield based on ^1^H NMR analysis: [moles of product per mol of aldehyde substrate] × 100%.

**Scheme 3:**
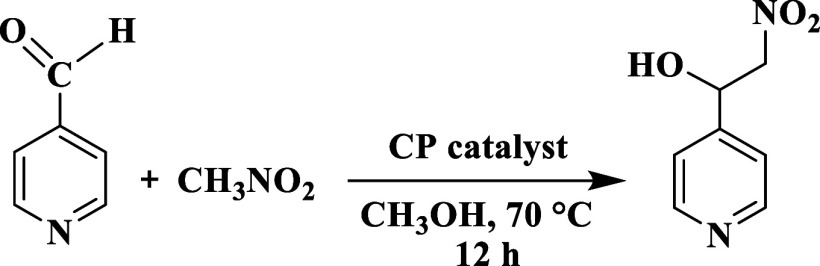
Pyridine-4-aldehyde Coupling with Nitromethane (Henry Reaction)

**Table 4:** Coupling of Pyridine-4-aldehyde with Nitromethane Catalyzed by **5** ^a^

entry	reaction time, min, T/°C	catalyst loading, mol %	solvent	product yield, %^b^
1	1, 70	4.0	CH_3_OH	48
2	2, 70	4.0	CH_3_OH	63
3	4, 70	4.0	CH_3_OH	74
4	6, 70	4.0	CH_3_OH	83
5	8, 70	4.0	CH_3_OH	91
6	10, 70	4.0	CH_3_OH	96
7	12, 70	4.0	CH_3_OH	99
8	16, 70	4.0	CH_3_OH	99
9	12, 25	4.0	CH_3_OH	18
10	12, 60	4.0	CH_3_OH	65
11	12, 80	4.0	CH_3_OH	86
12	12, 70	3.0	CH_3_OH	76
13	12, 70	5.0	CH_3_OH	99
14	12, 70	4.0	H_2_O	77
15	12, 70	4.0	CH_3_CN	17
16	12, 70	4.0	THF	54
17	12, 70	4.0	C_2_H_5_OH	51

a Conditions: pyridine-4-aldehyde (0.5 mmol), nitromethane (2.0 mmol), catalyst **5** (3–5 mol %), solvent (1.0 mL).

b Yield based on ^1^H NMR analysis: [moles of product per mol of aldehyde substrate] × 100%.

Among all the tested CPs, compound **5** revealed the highest catalytic activity in the conversion of pyridine-4-aldehyde to 2-nitro-1-(pyridin-4-yl)­ethan-1-ol with an almost quantitative ∼99% product yield (Table 3 and Figures S3 and S4). This might be explained by the presence of auxiliary H_2_biim ligands in **5**, in which the NH groups can facilitate base-catalyzed reactions.^39–42,54,55^ Hence, CP **5** was studied in detail to evaluate the various reaction parameters. The effect of reaction time shows that the product yield increases from 48 to 99% when the reaction is extended from 1 to 12 h (entries 1–7 in Table 4). Additionally, an increase of the catalyst loading from 3 to 5 mol % leads to the growth of product yield from 76 to 99% (entries 12–13, Table 4). We also screened alternative solvents, such as water, ethanol, acetonitrile, and THF, but these were less suitable compared to methanol, with yields ranging from 17 to 77%. In contrast to catalyst **5**, CPs **1**–**4** and **6**–**8** revealed lower activity that is still very appreciable with 78–93% maximum yields (Table 3). In particular, compounds **7** and **8** displayed product yields above 90%, thus suggesting that the CPs based on Mn­(II) and Co­(II) are also highly promising catalysts in the present type of reactions. Blank tests with H_4_cada (17% yield) or CdCl_2_ (14% yield) as catalysts showed significantly lower activity. Furthermore, in the absence of a catalyst, the product yield dropped to 3% (entries 9–11, Table 3).

To study the substrate scope, substituted aromatic aldehyde substrates were also screened. These experiments were carried out under optimum conditions, namely, with catalyst **5** (4.0 mol %), methanol solvent, and 12 h reaction time at 70 °C. The products were obtained with yields varying from 8 to 99% (Table 5). Benzaldehydes with strong electron-withdrawing groups (−NO_2_, −F, and −Cl) showed the highest reactivity and product yields, likely due to the enhanced electrophilicity of the substrates (entries 2–6, Table 5). However, benzaldehyde substrates with electron-donating groups (–Me and –OCH_3_) resulted in lower product yields (entries 9 and 10, Table 5). Also, in the reaction with 2-furaldehyde, a high product yield (94%) was obtained. In contrast, 1-naphthaldehyde and 9-anthraldehyde substrates revealed low product yields (23 and 8%, respectively; Table 5).

**Table 5:** Substrate Scope in the Coupling of Substituted Aromatic Aldehydes with Nitromethane Catalyzed by **5** ^a^

entry	substituted benzaldehyde	product yield, %^b^
1	benzaldehyde	84
2	2-nitrobenzaldehyde	93
3	3-nitrobenzaldehyde	95
4	4-nitrobenzaldehyde	96
5	4-chlorobenzaldehyde	83
6	4-fluorobenzaldehyde	95
7	pyridine-3-aldehyde	97
8	pyridine-4-aldehyde	98
9	4-methylbenzaldehyde	58
10	4-methoxybenzaldehyde	37
11	4-hydroxybenzaldehyde	21
12	2-thiophenecarboxaldehyde	76
13	2-furaldehyde	94
14	1-naphthaldehyde	23
15	9-anthraldehyde	8

a Conditions: aldehyde (0.5 mmol), nitromethane (2.0 mmol), catalyst **5** (4.0 mol %), methanol solvent (1.0 mL), 70 °C.

b Yield on the basis of ^1^H NMR analysis: [moles of product per mol of aldehyde substrate] × 100%.

The recyclability of **5** was also evaluated by running several reaction cycles with the same sample of the catalyst. In the end of each cycle, the catalyst was recovered by centrifugation, washed with methanol, and air-dried at ∼25 °C before being reused. The obtained results indicate that CP **5** maintains its activity for at least five cycles (Figure S5, Supporting Information), with yields of 98, 95, 94, and 92% achieved after the second through fifth runs, respectively. Additionally, PXRD patterns of the recovered catalyst confirm the structural stability of **5** (Figure S6, Supporting Information), although some minor new signals and peak broadening were observed. These changes are expected after catalyst reuse and are likely a result of impurities or a decrease in crystallinity. The catalytic performance of **5** is comparable to or even exceeds that of other CP-based heterogeneous catalytic systems (Table S3).^48–50^

## Conclusions

In the present work, we prepared eight new coordination polymers using 5,5′-(carbonylbis­(azanediyl))­diisophthalic acid (H_4_cada) as the primary linker. The obtained compounds **1**–**8** were fully characterized, and their structural features were investigated in detail. The structures of **1**–**8** range from 1D chains (**2**) and 2D layers (**1** and **3**–**6**) to 3D networks (**7**, **8**), in addition to revealing different topologies. The observed structural diversity arises from different coordination modes of the H_2_cada^2–^/cada^4–^ linkers, which, in turn, were influenced by the nature of the metal­(II) centers and the N-donor crystallization mediators.

All of the synthesized coordination polymers were also tested as heterogeneous catalysts for the C–C coupling reaction of pyridine-4-aldehyde (model substrate) with nitromethane. Substrate scope and the effects of reaction time, temperature, solvent type, catalyst loading, and recycling were explored, allowing identification of highly efficient (up to 99% product yields), selective, and recyclable catalytic systems. In particular, CP **5** was identified as the most promising catalyst.

This work widens a limited family of CPs derived from H_4_cada, illustrating the versatility of this urea-diisophthalate linker in the hydrothermal synthesis of coordination polymers. Further exploration of this and related carboxylate linkers in the synthesis of functional coordination polymers^51,52^ is currently in progress in our laboratories.

## Supplementary Material



## Notes

Synthesis and analytical data for **1**–**8**, FTIR spectra, PXRD patterns, additional catalysis data, bonding parameters, and crystallographic data in CIF format (CCDC-2468942–2468949) (PDF)
