# Supplementary material for: Coordination Polymers Driven by Urea-Diisophthalate Linkers: From Hydrothermal Assembly and Structural Diversity to Catalytic Applications
Source: Inorg Chem. 2025 Aug 26;64(35):17960–71. doi: 10.1021/acs.inorgchem.5c03114 (PMC12818755; doi:10.1021/acs.inorgchem.5c03114)
Supplement: Supplementary file 1 [file ic5c03114_si_001.pdf]

Supporting Information

Coordination Polymers Driven by Urea-Diisophthalate Linkers: From Hydrothermal Assembly and Structural Diversity to Catalytic Applications

Yu Chen,[#] Xiaoxiang Fan,[§] Hongyu Wang,[#,*] Jinzhong Gu,[#,*] Marina V. Kirillova,[†] and Alexander M. Kirillov[†,*]

[#]*State Key Laboratory of Natural Product Chemistry, College of Chemistry and Chemical Engineering, Lanzhou University, Lanzhou 730000, People's Republic of China*

[§]*Nuclear Power Institute of China, Chengdu 610041, People's Republic of China*

[†]*MINDlab: Molecular Design & Innovation Laboratory, Centro de Química Estrutural, Institute of Molecular Sciences, Departamento de Engenharia Química, Instituto Superior Técnico, Universidade de Lisboa, Av. Rovisco Pais, 1049-001, Lisbon, Portugal*

[*]Corresponding author, E-mail: hywang@lzu.edu.cn (H.-Y. Wang), gujzh@lzu.edu.cn (J.-Z. Gu), kirillov@tecnico.ulisboa.pt (A. M. Kirillov).

Supporting Information contains:

Synthesis and analytical data for 1–8.

Figure S1 FTIR spectra of compounds **1**−**8**.

Figure S2 Powder X-ray diffraction patterns of **1**−**8**.

Figure S3 Typical ^{1}H NMR spectrum and its integration for determining catalytic reaction products.

Figure S4 Accumulation of 2-nitro-1-(pyridin-4-yl)ethan-1-ol vs. time in the catalytic reaction.

Figure S5 Catalyst recycling experiments in the coupling of pyridine-4-aldehyde with nitromethane catalyzed by **5**.

Figure S6 PXRD patterns of **5**.

Table S1 Selected bond lengths and bond angles for compounds **1**–**8**.

Table S2 Hydrogen bonds in crystal packing of compounds **1**–**8**.

Table S3 Comparison of various catalysts in the coupling reactions of aldehydes with nitromethane.

Materials and Methods

All chemicals and solvents were obtained from commercial suppliers. 5,5'-(carbonylbis(azanediyl))diisophthalic acid (H_4cada) was acquired from Yanshen Technology Co., Ltd. C/N/H analyses were run on an Elementar Vario EL elemental analyzer. Bruker EQUINOX 55 spectrometer was used for recording the FTIR spectra (KBr discs). LINSEIS STA PT1600 thermal analyzer was used for thermogravimetric (TGA) measurements (heating rate: 10°C/min; N_2 flow). PXRD (powder X-ray diffraction) analyses were carried out on a Rigaku-Dmax 2400 diffractometer (Cu-Kα radiation, λ = 1.54060 Å). Solution ^{1}H NMR spectra were recorded on a JNM ECS 400 M spectrometer.

Synthesis and analytical data for 1–8

Synthesis of [Mn(μ₃-H₂cada)(phen)(H₂O)]ₙ·2nH₂O (1). A mixture of $MnCl_2·4H_2O$ (39.6 mg, 0.20 mmol), H_4cada (77.6 mg, 0.20 mmol), phen (39.6 mg, 0.20 mmol), NaOH (16.0 mg, 0.40 mmol), and H_2O (10 mL) was stirred at room temperature for 15 min, then sealed in a 25 mL Teflon-lined stainless steel vessel, and heated at 160 °C for 3 days, followed by cooling to room temperature at a rate of 10 °C·h^{-1}. Colorless block-shaped crystals of **1** were isolated manually, washed with distilled water and dried (yield: 43% based on H_4cada). Anal. Calcd for $C_{29}H_{24}MnN_4O_{12}$: C, 51.57; H, 3.58; N, 8.29. Found: C, 51.81; H, 3.55; N, 8.35%. FTIR (KBr, cm^{-1}): 3398 w, 3331 w, 3058 w, 1709 m, 1625 w, 1579 s, 1550 s, 1516 w, 1424 m, 1394 w, 1361 w, 1318 w, 1264 w, 1205 w, 1142 w, 1100 w, 1041 w, 995 w, 903 w, 848 m, 782 m, 727 m, 634 w.

Synthesis of [Mn₂(μ₄-cada)(phen)₃(H₂O)]ₙ·2nH₂O (2). A mixture of $MnCl_2·4H_2O$ (39.6 mg, 0.20 mmol), H_4cada (38.8 mg, 0.10 mmol), phen (39.6 mg, 0.20 mmol), NaOH (16.0 mg, 0.40 mmol), and H_2O (10 mL) was stirred at room temperature for 15 min, then sealed in a 25 mL Teflon-lined stainless steel vessel, and heated at 160 °C for 3 days, followed by cooling to room temperature at a rate of 10 °C·h^{-1}. Yellow block-shaped crystals of **2** were isolated manually, washed with distilled water and dried (yield: 45% based on H_4cada). Anal. Calcd for $C_{53}H_{38}Mn_2N_8O_{12}$: C, 58.47; H, 3.52; N, 10.29. Found: C, 58.73; H, 3.50; N, 10.43%. FTIR (KBr, cm^{-1}): 3335 w, 3086 w, 2818 w, 1701 w, 1625 m, 1579 s, 1550 s, 1512

w, 1420 m, 1361 m, 1323 w, 1260 w, 1202 m, 1147 w, 1101 w, 1041 w, 1000 w, 928 w, 845 m, 782 m, 727 m, 635 w.

Synthesis of $[Mn_2(\mu_5\text{-}cada)(bipy)_2(H_2O)_2]_n{\cdot}3nH_2O$ *(3).* A mixture of $MnCl_2{\cdot}4H_2O$ (39.6 mg, 0.20 mmol), H_4cada (38.8 mg, 0.10 mmol), bipy (31.2 mg, 0.20 mmol), NaOH (16.0 mg, 0.40 mmol), and H_2O (10 mL) was stirred at room temperature for 15 min, then sealed in a 25 mL Teflon-lined stainless steel vessel, and heated at 160 °C for 3 days, followed by cooling to room temperature at a rate of 10 $°C{\cdot}h^{-1}$. Yellow block-shaped crystals of **3** were isolated manually, washed with distilled water and dried (yield: 43% based on H_4cada). Anal. Calcd for $C_{37}H_{34}Mn_2N_6O_{14}$: C, 49.57; H, 3.82; N, 9.37. Found: C, 49.36; H, 3.84; N, 9.54%. FTIR (KBr, cm^{-1}): 3339 w, 1705 w, 1625 w, 1592 m, 1554 s, 1474 w, 1441 m, 1420 w, 1374 s, 1318 w, 1251 w, 1210 w, 1151 w, 1109 w, 1058 w, 1021 w, 906 w, 911 w, 782 m, 756 w, 718 w, 688 w, 651 w, 534 w.

Synthesis of $[Cd_2(\mu_5\text{-}cada)(bipy)_2(H_2O)_2]_n{\cdot}3nH_2O$ *(4).* A mixture of $CdCl_2{\cdot}H_2O$ (40.2 mg, 0.20 mmol), H_4cada (38.8 mg, 0.10 mmol), bipy (31.2 mg, 0.20 mmol), NaOH (16.0 mg, 0.40 mmol), and H_2O (10 mL) was stirred at room temperature for 15 min, then sealed in a 25 mL Teflon-lined stainless steel vessel, and heated at 160 °C for 3 days, followed by cooling to room temperature at a rate of 10 $°C{\cdot}h^{-1}$. Colorless block-shaped crystals of **4** were isolated manually, washed with distilled water and dried (yield: 46% based on H_4cada). Anal. Calcd for $C_{37}H_{34}Cd_2N_6O_{14}$: C, 43.93; H, 3.39; N, 8.31. Found: C, 43.71; H, 3.37; N, 8.57%. FTIR (KBr, cm^{-1}): 3314 w, 3028 w, 2780 w, 1752 w, 1722 m, 1554 m, 1474 w, 1436 m, 1412 w, 1374 s, 1319 w, 1248 w, 1210 w, 1159 w, 1105 w, 1055 w, 1021 w, 966 w, 898 w, 782 m, 756 m, 722 w, 647 w, 534 w.

Synthesis of $[Cd_2(\mu_5\text{-}cada)(H_2biim)_3]_n{\cdot}2nH_2O$ *(5).* A mixture of $CdCl_2{\cdot}H_2O$ (40.2 mg, 0.20 mmol), H_4cada (38.8 mg, 0.10 mmol), H_2biim (26.8 mg, 0.20 mmol), NaOH (16.0 mg, 0.40 mmol), and H_2O (10 mL) was stirred at room temperature for 15 min, then sealed in a 25 mL Teflon-lined stainless steel vessel, and heated at 160 °C for 3 days, followed by cooling to room temperature at a rate of 10 $°C{\cdot}h^{-1}$. Colorless block-shaped crystals of **5** were isolated manually, washed with distilled water and dried (yield: 32% based on H_4cada). Anal. Calcd for $C_{35}H_{30}Cd_2N_{14}O_{11}$: C, 40.13; H, 2.89; N, 18.72. Found: C, 40.38; H, 2.87; N, 18.51%. FTIR (KBr, cm^{-1}): 3268 w, 3129 w, 2995 w, 2911 w, 2768 w, 1781 w, 1701 w, 1617 w,

1550 s, 1525 w, 1424 w, 1357 s, 1315 m, 1265 w, 1218 w, 1180 w, 1121 w, 1016 w, 987 w, 962 w, 920 w, 865 w, 777 m, 752 w, 727 w, 684 w, 588 w, 546 w.

Synthesis of $[H_2bpa]_n[Mn(\mu_3\text{-}cada)(H_2O)_2]_n\cdot 3nH_2O$ ***(6)***. A mixture of $MnCl_2\cdot 4H_2O$ (39.6 mg, 0.20 mmol), H_4cada (38.8 mg, 0.10 mmol), bpa (34.2 mg, 0.20 mmol), NaOH (16.0 mg, 0.40 mmol), and H_2O (10 mL) was stirred at room temperature for 15 min, then sealed in a 25 mL Teflon-lined stainless steel vessel, and heated at 160 °C for 3 days, followed by cooling to room temperature at a rate of 10 $°C\cdot h^{-1}$. Yellow block-shaped crystals of **6** were isolated manually, washed with distilled water and dried (yield: 41% based on H_4cada). Anal. Calcd for $C_{27}H_{29}MnN_5O_{14}$: C, 46.16; H, 4.16; N, 9.97. Found: C, 46.43; H, 4.13; N, 10.18%. FTIR (KBr, cm^{-1}): 3468 w, 3104 w, 2944 w, 2835 w, 2659 w, 1688 w, 1630 s, 1567 m, 1533 w, 1441 w, 1407 w, 1369 m, 1344 w, 1272 w, 1235 w, 1205 w, 1105 w, 1075 w, 995 w, 916 w, 848 w, 819 w, 768 w, 718 w, 567 w.

Synthesis of $[Co_2(\mu_6\text{-}cada)(\mu\text{-}dpey)_{0.5}(H_2O)_4]_n\cdot 2nH_2O$ *(7)*. A mixture of $CoCl_2\cdot 6H_2O$ (47.6 mg, 0.20 mmol), H_4cada (38.8 mg, 0.10 mmol), dpey (36.4 mg, 0.20 mmol), NaOH (16.0 mg, 0.40 mmol), and H_2O (10 mL) was stirred at room temperature for 15 min, then sealed in a 25 mL Teflon-lined stainless steel vessel, and heated at 160 °C for 3 days, followed by cooling to room temperature at a rate of 10 $°C\cdot h^{-1}$. Pink block-shaped crystals of **7** were isolated manually, washed with distilled water and dried (yield: 44% based on H_4cada). Anal. Calcd for $C_{23}H_{25}Co_2N_3O_{15}$: C, 39.39; H, 3.59; N, 5.99. Found: C, 39.63; H, 3.62; N, 5.94%. FTIR (KBr, cm^{-1}): 3209 w, 2772 w, 1739 w, 1705 w, 1617 s, 1571 s, 1419 m, 1374 s, 1327 w, 1264 w, 1222 w, 1071 w, 1024 w, 962 w, 899 w, 840 w, 772 m, 718 m, 672w, 555 w.

Synthesis of $[Mn_2(\mu_6\text{-}cada)(\mu\text{-}dpea)_{0.5}(H_2O)_4]_n\cdot 2nH_2O$ ***(8)***. A mixture of $MnCl_2\cdot 4H_2O$ (39.6 mg, 0.20 mmol), H_4cada (38.8 mg, 0.10 mmol), dpea (36.8 mg, 0.20 mmol), and H_2O (10 mL) was stirred at room temperature for 15 min, then sealed in a 25 mL Teflon-lined stainless steel vessel, and heated at 160 °C for 3 days, followed by cooling to room temperature at a rate of 10 $°C\cdot h^{-1}$. Yellow block-shaped crystals of **8** were isolated manually, washed with distilled water and dried (yield: 47% based on H_4cada). Anal. Calcd for $C_{46}H_{52}Mn_4N_6O_{30}$: C, 39.79; H, 3.77; N, 6.05. Found: C, 39.53; H, 3.75; N, 6.10%. FTIR (KBr, cm^{-1}): 3590 w, 3316 w, 1731 w, 1698 w, 1619 m, 1565 s, 1437 m, 1371 s, 1258 w, 1217 w, 1067 w, 1022 w, 967 w, 897 w, 839 w, 806 w, 781 w, 719 w, 615 w, 552 w.

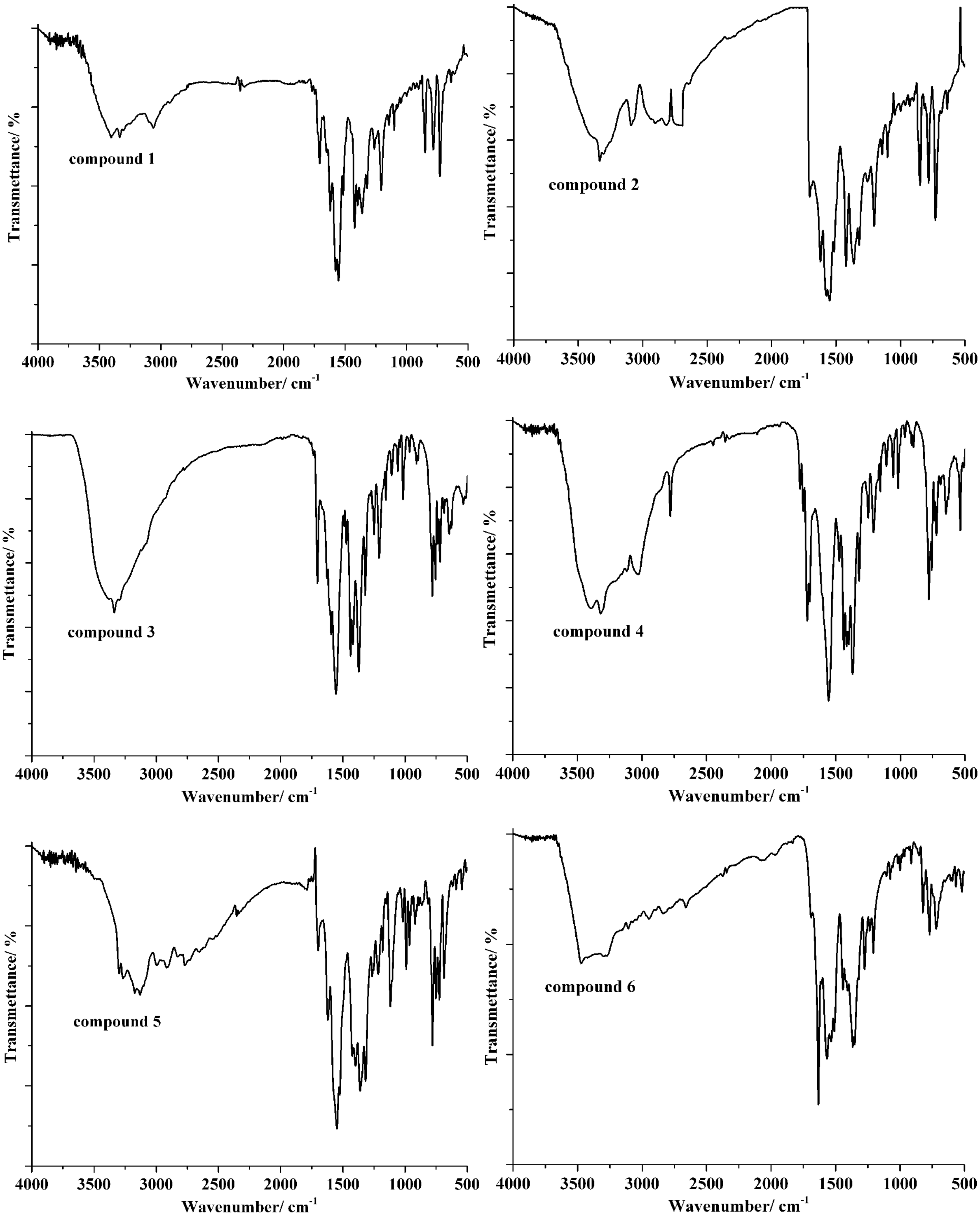
compound 1
compound 2
compound 3
compound 4
compound 5
compound 6
Transmittance/ %
Wavenumber/ cm^{-1}
4000 3500 3000 2500 2000 1500 1000 500

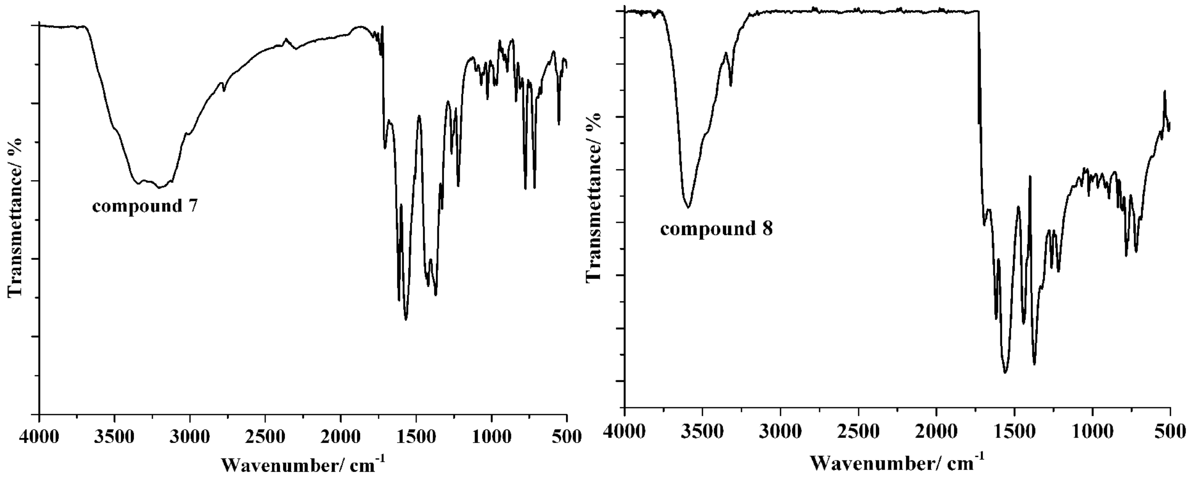

Figure S1. FTIR spectra of compounds **1–8**.

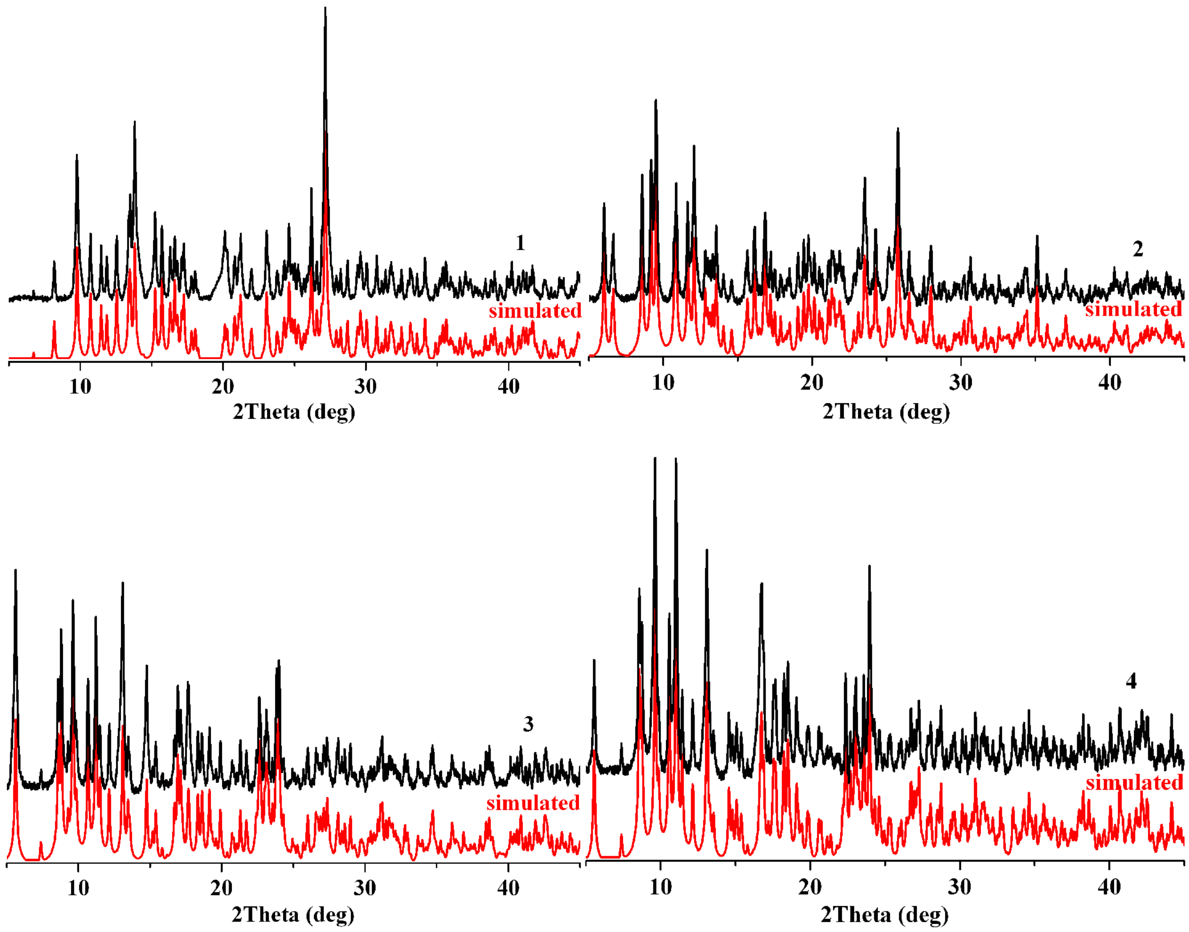

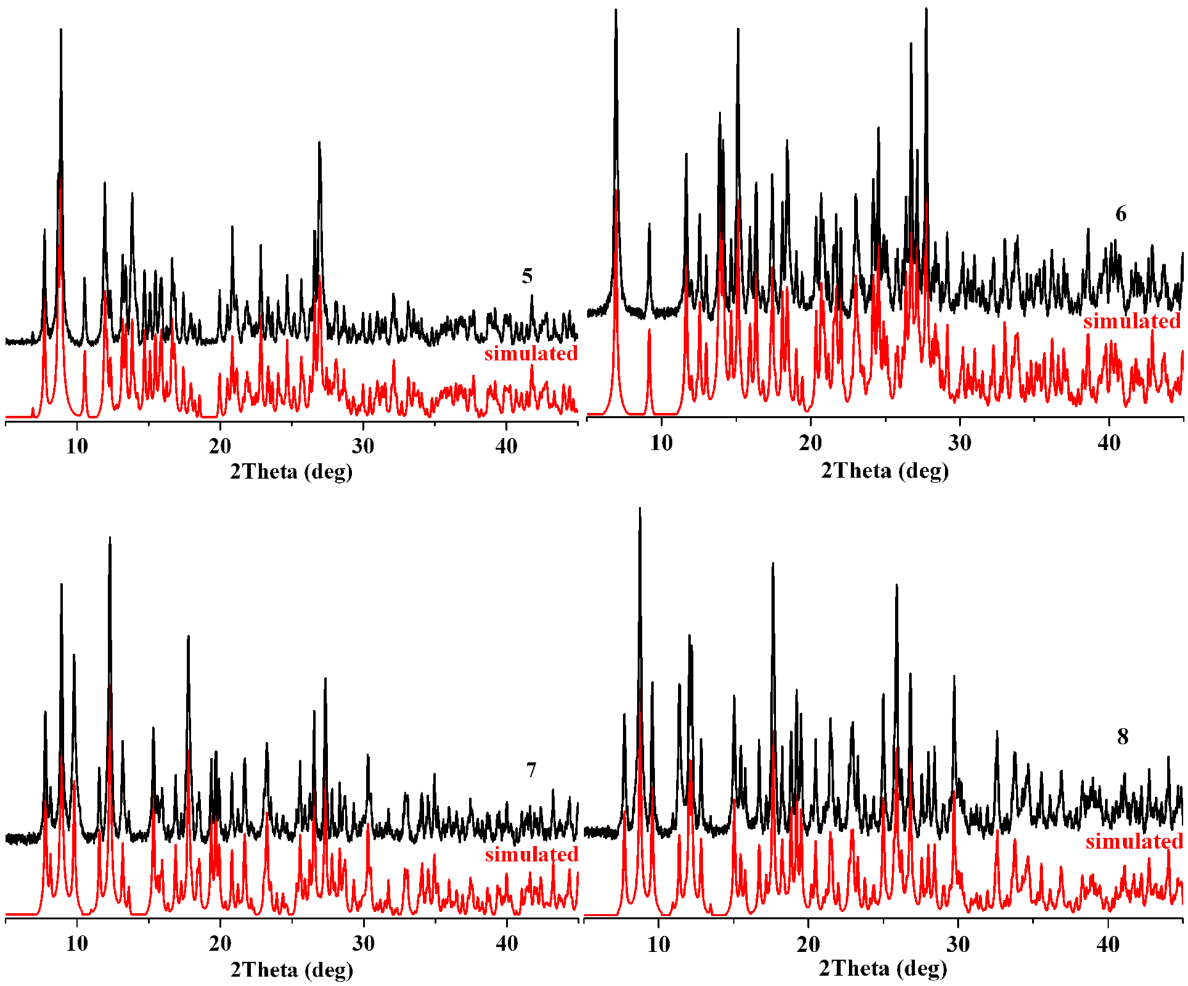

Figure S2. PXRD patterns of **1**–**8** at room temperature.

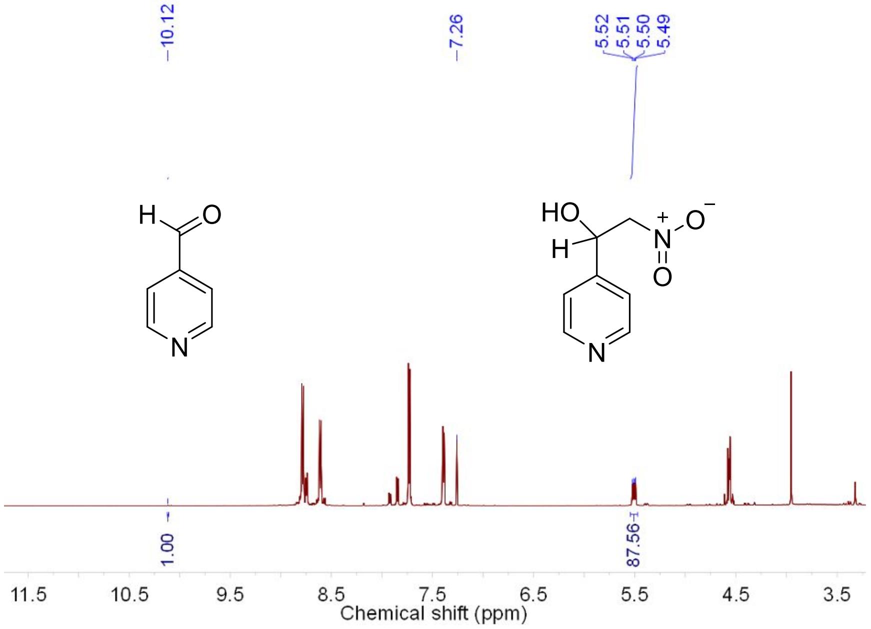

Figure S3. Typical ^{1}H NMR spectrum and its integration for the determination of catalytic reaction product (conditions of Table 3, Entry 7).

Calculation of the product yield based on the data of Figure S3

Yield:

The C(=O)H signal of pyridine-4-aldehyde (substrate) appears at δ 10.12 ppm, while 2-nitro-1-(pyridin-4-yl)ethan-1-ol (product) shows a characteristic signal at δ 5.49-5.52 ppm.

Total amount of compounds: pyridine-4-aldehyde + product = 1 + 87.56 = 88.56.

Percentage of the unreacted pyridine-4-aldehyde: (1/88.56) × 100 = 1.129%.

Conversion of pyridine-4-aldehyde = yield of product = 100−1.129= 98.87%.

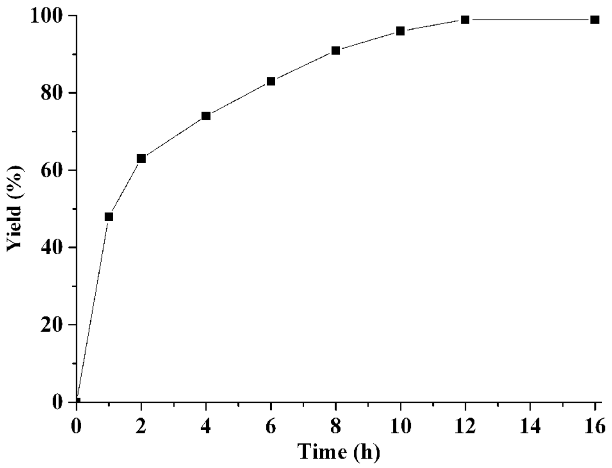

Figure S4. Accumulation of 2-nitro-1-(pyridin-4-yl)ethan-1-ol vs. time in the Henry reaction of pyridine-4-aldehyde with nitromethane catalyzed by **5**. Reaction conditions are those of Table 3, entries 1−8.

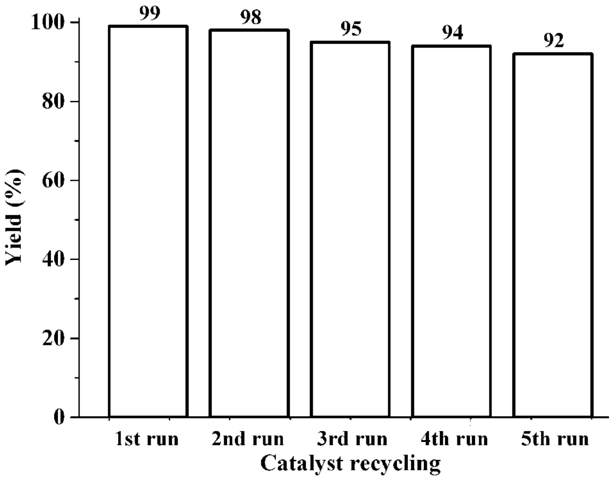

Figure S5. Catalyst recycling experiments in the coupling of pyridine-4-aldehyde with nitromethane catalyzed by **5**. Reaction conditions are those of Table 3, entry 7.

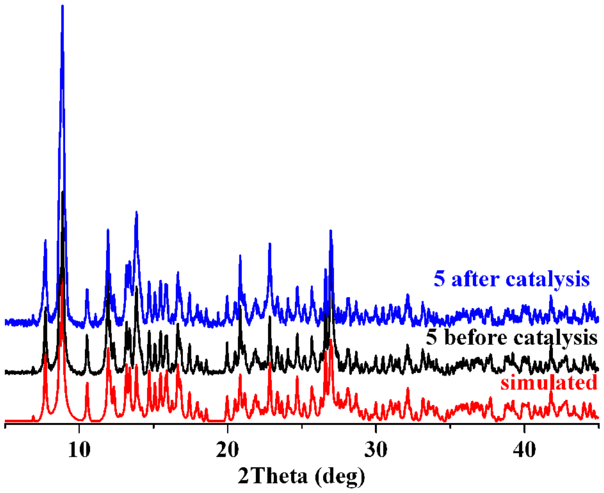

Figure S6. PXRD patterns of **5**: simulated (red), before (black) and after (blue) catalysis.

Table S1. Selected bond lengths [Å] and angles [°] for **1–8**[a].

**1**					
Mn(1)-O(1)	2.122(2)	Mn(1)-O(4)i	2.161(2)	Mn(1)-O(7)ii	2.126(2)
Mn(1)-O(10)	2.280(2)	Mn(1)-N(3)	2.303(3)	Mn(1)-N(4)	2.242(3)
O(1)-Mn(1)-O(7)ii	88.47(9)	O(1)-Mn(1)-O(4)i	95.07(9)	O(7)ii-Mn(1)-O(4)i	101.98(9)
O(1)-Mn(1)-N(4)	91.50(9)	O(7)ii-Mn(1)-N(4)	91.94(9)	N(4)-Mn(1)-O(4)i	164.73(9)
O(1)-Mn(1)-O(10)	167.06(9)	O(7)ii-Mn(1)-O(10)	78.59(9)	O(4)i-Mn(1)-O(10)	87.85(9)
O(10)-Mn(1)-N(4)	88.81(9)	O(1)-Mn(1)-N(3)	109.72(9)	O(7)ii-Mn(1)-N(3)	156.30(9)
O(4)i-Mn(1)-N(3)	91.70(9)	N(3)-Mn(1)-N(4)	73.10(9)	O(10)-Mn(1)-N(3)	82.74(9)
**2**					
Mn(1)-O(1)	2.1108(15)	Mn(1)-O(7)i	2.1295(16)	Mn(1)-O(8)ii	2.3751(16)
Mn(1)-O(9)ii	2.2269(15)	Mn(1)-N(3)	2.3204(18)	Mn(1)-N(4)	2.295(2)
Mn(2)-O(4)	2.1133(17)	Mn(2)-O(10)	2.1313(16)	Mn(2)-N(5)	2.262(2)
Mn(2)-N(6)	2.335(2)	Mn(2)-N(7)	2.247(2)	Mn(2)-N(8)	2.3636(19)
O(1)-Mn(1)-O(7)i	97.62(7)	O(1)-Mn(1)-O(9)ii	124.53(6)	O(7)i-Mn(1)-O(9)ii	93.64(6)
O(1)-Mn(1)-N(4)	94.76(7)	O(7)i-Mn(1)-N(4)	163.07(7)	O(9)ii-Mn(1)-N(4)	88.77(7)
O(1)-Mn(1)-N(3)	86.19(6)	O(7)i-Mn(1)-N(3)	97.27(6)	O(9)ii-Mn(1)-N(3)	145.62(6)
N(4)-Mn(1)-N(3)	72.03(7)	O(1)-Mn(1)-O(8)ii	173.51(7)	O(7)i-Mn(1)-O(8)ii	88.58(6)
O(8)ii-Mn(1)-O(9)ii	56.56(5)	O(8)ii-Mn(1)-N(4)	78.77(7)	O(8)ii-Mn(1)-N(3)	91.14(6)
O(4)-Mn(2)-O(10)	87.25(7)	O(4)-Mn(2)-N(7)	100.21(7)	O(10)-Mn(2)-N(7)	97.29(8)
O(4)-Mn(2)-N(5)	101.63(7)	O(10)-Mn(2)-N(5)	104.08(7)	N(7)-Mn(2)-N(5)	149.97(8)
O(4)-Mn(2)-N(6)	168.77(7)	O(10)-Mn(2)-N(6)	85.00(7)	N(6)-Mn(2)-N(7)	88.83(7)
N(5)-Mn(2)-N(6)	72.52(8)	O(4)-Mn(2)-N(8)	85.69(6)	O(10)-Mn(2)-N(8)	165.99(7)
N(7)-Mn(2)-N(8)	72.15(7)	N(5)-Mn(2)-N(8)	89.20(7)	N(6)-Mn(2)-N(8)	103.56(7)
**3**					
Mn(1)-O(1)	2.1024(18)	Mn(1)-O(2)i	2.1087(19)	Mn(1)-O(8)ii	2.2172(19)
Mn(1)-O(10)	2.226(2)	Mn(1)-N(3)	2.287(2)	Mn(1)-N(4)	2.325(2)
Mn(2)-O(3)iii	2.2235(19)	Mn(2)-O(4)iii	2.417(2)	Mn(2)-O(6)	2.4267(19)
Mn(2)-O(7)	2.2311(19)	Mn(2)-O(11)	2.191(2)	Mn(2)-N(5)	2.250(2)
Mn(2)-N(6)	2.261(2)				
O(1)-Mn(1)-O(2)i	108.48(8)	O(1)-Mn(1)-O(8)ii	99.03(7)	O(2)i-Mn(1)-O(8)ii	81.56(8)
O(1)-Mn(1)-O(10)	88.45(8)	O(10)-Mn(1)-O(2)i	159.94(8)	O(8)ii-Mn(1)-O(10)	85.28(7)
O(1)-Mn(1)-N(3)	154.51(8)	O(2)i-Mn(1)-N(3)	86.61(9)	O(8)ii-Mn(1)-N(3)	103.55(8)

O(10)-Mn(1)-N(3)	81.81(8)	O(1)-Mn(1)-N(4)	85.53(8)	O(2)i-Mn(1)-N(4)	101.55(10)
O(8)ii-Mn(1)-N(4)	173.44(9)	O(10)-Mn(1)-N(4)	90.15(9)	N(3)-Mn(1)-N(4)	71.07(9)
O(3)iii-Mn(2)-O(11)	78.60(8)	O(7)-Mn(2)-O(11)	94.02(8)	O(3)iii-Mn(2)-O(7)	137.95(7)
O(11)-Mn(2)-N(5)	100.03(9)	O(3)iii-Mn(2)-N(5)	87.39(8)	O(7)-Mn(2)-N(5)	134.53(8)
O(11)-Mn(2)-N(6)	165.77(9)	O(3)iii-Mn(2)-N(6)	112.60(8)	O(7)-Mn(2)-N(6)	83.34(8)
N(5)-Mn(2)-N(6)	72.69(9)	O(4)iii-Mn(2)-O(11)	100.91(8)	O(4)iii-Mn(2)-O(3)iii	55.86(7)
O(4)iii-Mn(2)-O(7)	85.94(7)	O(4)iii-Mn(2)-N(5)	132.21(8)	O(4)iii-Mn(2)-N(6)	92.87(8)
O(11)-Mn(2)-O(6)	84.80(8)	O(3)iii-Mn(2)-O(6)	158.60(7)	O(7)-Mn(2)-O(6)	56.09(7)
N(5)-Mn(2)-O(6)	82.27(8)	N(6)-Mn(2)-O(6)	82.13(8)	O(4)iii-Mn(2)-O(6)	142.00(7)
**4**					
Cd(1)-O(1)i	2.2483(19)	Cd(1)-O(2)	2.2174(19)	Cd(1)-O(7)ii	2.3355(18)
Cd(1)-O(10)	2.3283(19)	Cd(1)-N(3)	2.346(2)	Cd(1)-N(4)	2.389(2)
Cd(2)-O(3)iii	2.5055(17)	Cd(2)-O(4)iii	2.3141(19)	Cd(2)-O(8)	2.3077(19)
Cd(2)-O(9)	2.5487(19)	Co(2)-O(11)	2.307(2)	Cd(2)-N(5)	2.327(2)
Cd(2)-N(6)	2.343(3)				
O(2)-Cd(1)-O(1)i	110.41(7)	O(2)-Cd(1)-O(10)	87.85(7)	O(1)i-Cd(1)-O(10)	156.01(7)
O(7)i-Cd(1)-O(2)	102.50(7)	O(1)i-Cd(1)-O(7)ii	76.79(7)	O(10)-Cd(1)-O(7)i	84.31(7)
N(3)-Cd(1)-O(2)	152.59(7)	O(1)i-Cd(1)-N(3)	87.27(8)	N(3)-Cd(1)-O(10)	82.25(8)
O(7)ii-Cd(1)-N(3)	101.87(7)	O(2)-Cd(1)-N(4)	84.42(8)	N(4)-Cd(1)-O(1)i	107.16(9)
N(4)-Cd(1)-O(10)	89.48(8)	O(7)i-Cd(1)-N(4)	170.49(7)	N(3)-Cd(1)-N(4)	70.06(9)
O(8)-Cd(2)-O(11)	94.20(8)	O(8)-Cd(2)-O(4)iii	137.48(6)	O(4)iii-Cd(2)-O(11)	77.31(8)
O(8)-Cd(2)-N(5)	133.23(7)	O(11)-Cd(2)-N(5)	101.44(9)	N(5)-Cd(2)-O(4)iii	89.15(8)
O(8)-Cd(2)-N(6)	82.98(8)	N(6)-Cd(2)-O(11)	164.68(8)	N(6)-Cd(2)-O(4)iii	114.77(8)
N(5)-Cd(2)-N(6)	70.59(9)	O(8)-Cd(2)-O(3)iii	87.83(6)	O(11)-Cd(2)-O(3)iii	99.85(7)
O(3)iii-Cd(2)-O(4)iii	53.89(6)	O(3)iii-Cd(2)-N(5)	131.17(7)	O(3)iii-Cd(2)-N(6)	95.11(7)
O(8)-Cd(2)-O(9)	53.55(6)	O(9)-Cd(2)-O(11)	86.14(8)	O(9)-Cd(2)-O(4)iii	160.28(7)
N(5)-Cd(2)-O(9)	83.64(7)	O(9)-Cd(2)-N(6)	80.06(8)	O(3)iii-Cd(2)-O(9)	141.34(6)
**5**					
Cd(1)-O(2)	2.167(3)	Cd(1)-O(3)ii	2.826(2)	Cd(1)-O(8)i	2.198(3)
Cd(1)-O(9)i	2.722(3)	Cd(1)-N(3)	2.365(4)	Cd(1)-N(6)	2.246(3)
Cd(2)-O(4)	2.481(3)	Cd(2)-O(7)iii	2.475(3)	Cd(2)-N(7)	2.279(3)
Cd(2)-N(10)	2.317(3)	Cd(2)-N(11)	2.281(3)	Cd(2)-N(14)	2.354(3)
O(2)-Cd(1)-O(8)i	89.09(11)	O(2)-Cd(1)-N(6)	131.38(11)	N(6)-Cd(1)-O(8)i	138.25(11)
O(2)-Cd(1)-N(3)	103.55(13)	N(3)-Cd(1)-O(8)i	108.84(12)	N(6)-Cd(1)-N(3)	75.29(12)
O(2)-Cd(1)-O(3)ii	76.39(11)	O(8)i-Cd(1)-O(3)ii	111.31(11)	O(9)i-Cd(1)-O(3)ii	120.00(12)
N(3)-Cd(1)-O(3)ii	139.78(12)	N(6)-Cd(1)-O(3)ii	76.33(12)	O(2)-Cd(1)-O(9)i	140.42(12)
O(8)i-Cd(1)-O(9)i	51.71(12)	N(3)-Cd(1)-O(9)i	86.90(12)	N(6)-Cd(1)-O(9)i	88.15(13)
N(11)-Cd(2)-N(7)	174.69(13)	N(7)-Cd(2)-N(10)	75.92(11)	N(11)-Cd(2)-N(10)	100.94(12)
N(14)-Cd(2)-N(7)	109.22(12)	N(11)-Cd(2)-N(14)	74.17(12)	N(11)-Cd(2)-N(14)	173.98(12)
N(7)-Cd(2)-O(7)iii	90.37(11)	N(11)-Cd(2)-O(7)iii	85.46(11)	N(10)-Cd(2)-O(7)iii	92.41(11)
N(14)-Cd(2)-O(7)iii	90.71(11)	N(7)-Cd(2)-O(4)	79.65(11)	N(11)-Cd(2)-O(4)	104.62(11)
O(4)-Cd(2)-N(10)	87.15(11)	N(14)-Cd(2)-O(4)	90.68(11)	O(4)-Cd(2)-O(7)iii	169.82(9)
**6**					
Mn(1)-O(1)	2.0844(15)	Mn(1)-O(7)i	2.1615(16)	Mn(1)-O(8)ii	2.2057(15)
Mn(1)-O(9)ii	2.3417(14)	Mn(1)-O(10)	2.1644(17)	Mn(1)-O(11)	2.1942(18)
O(1)-Mn(1)-O(7)i	97.28(6)	O(1)-Mn(1)-O(10)	92.49(7)	O(7)i-Mn(1)-O(10)	95.74(7)
O(1)-Mn(1)-O(11)	92.83(7)	O(7)i-Mn(1)-O(11)	169.42(7)	O(10)-Mn(1)-O(11)	86.90(7)
O(1)-Mn(1)-O(8)ii	97.60(6)	O(8)ii-Mn(1)-O(7)i	88.51(6)	O(8)ii-Mn(1)-O(10)	168.48(7)
O(8)ii-Mn(1)-O(11)	87.05(6)	O(9)ii-Mn(1)-O(1)	154.94(6)	O(9)ii-Mn(1)-O(7)i	86.04(6)
O(9)ii-Mn(1)-O(10)	111.96(6)	O(9)ii-Mn(1)-O(11)	83.48(6)	O(9)ii-Mn(1)-O(8)ii	57.54(5)

**7**					
Co(1)-O(1)	2.0611(17)	Co(1)-O(7)ii	2.1257(16)	Co(1)-O(9)iii	2.1023(17)
Co(1)-O(10)	2.102(2)	Co(1)-O(11)	2.136(2)	Co(1)-O(12)	2.081(2)
Co(2)-O(2)	2.0011(16)	Co(2)-O(3)i	2.188(2)	Co(2)-O(8)iii	2.0025(16)
Ni(2)-O(10)	2.0519(16)	Ni(2)-N(5)	2.1032(17)	Ni(2)-N(6)iv	2.1237(17)
Co(2)-O(13)	2.110(2)	Co(2)-N(3)	2.097(2)		
O(1)-Co(1)-O(12)	83.43(9)	O(1)-Co(1)-O(9)iii	96.96(7)	O(9)iii-Co(1)-O(12)	92.51(9)
O(1)-Co(1)-O(10)	170.11(9)	O(10)-Co(1)-O(12)	93.55(12)	O(9)iii-Co(1)-O(10)	92.57(9)
O(1)-Co(1)-O(7)ii	89.62(7)	O(7)ii-Co(1)-O(12)	92.12(8)	O(7)ii-Co(1)-O(9)iii	172.35(8)
O(10)-Co(1)-O(7)ii	81.06(8)	O(1)-Co(1)-O(11)	87.41(8)	O(11)-Co(1)-O(12)	170.84(8)
O(11)-Co(1)-O(9)iii	88.28(8)	O(11)-Co(1)-O(10)	95.54(12)	O(11)-Co(1)-O(7)ii	88.12(8)
O(2)-Co(2)-O(8)iii	169.45(7)	N(3)-Co(2)-O(8)iii	96.23(8)	O(2)-Co(2)-N(3)	93.79(8)
O(13)-Co(2)-O(8)iii	91.33(8)	O(2)-Co(2)-O(13)	91.14(8)	N(3)-Co(2)-O(13)	94.72(8)
O(3)i-Co(2)-O(8)iii	84.76(7)	O(2)-Co(2)-O(3)i	89.88(8)	N(3)-Co(2)-O(3)i	102.34(8)
**8**					
Mn(1)-O(2)	2.1498(18)	Mn(1)-O(4)i	2.2127(17)	Mn(1)-O(7)ii	2.1422(17)
Mn(1)-O(9)	2.231(2)	Mn(1)-O(10)	2.185(2)	Mn(1)-O(11)	2.170(2)
Mn(2)-O(1)ii	2.1084(17)	Mn(2)-O(5)iii	2.2454(19)	Mn(2)-O(8)	2.0899(17)
Mn(2)-O(12)	2.187(2)	Mn(2)-N(3)	2.193(2)		
O(2)-Mn(1)-O(7)ii	97.98(8)	O(11)-Mn(1)-O(7)ii	81.31(8)	O(2)-Mn(1)-O(11)	93.27(8)
O(10)-Mn(1)-O(7)ii	170.00(11)	O(2)-Mn(1)-O(10)	91.00(9)	O(10)-Mn(1)-O(11)	93.85(14)
O(4)i-Mn(1)-O(7)ii	91.97(7)	O(2)-Mn(1)-O(4)i	168.91(7)	O(11)-Mn(1)-O(4)i	93.08(8)
O(4)i-Mn(1)-O(10)	79.50(9)	O(7)ii-Mn(1)-O(9)	86.23(8)	O(2)-Mn(2)-O(9)	88.70(8)
O(9)-Mn(1)-O(11)	167.53(8)	O(9)-Mn(1)-O(10)	98.43(14)	O(4)i-Mn(2)-O(9)	87.06(7)
O(1)ii-Mn(2)-O(8)	168.35(7)	O(12)-Mn(2)-O(8)	92.50(8)	O(1)ii-Mn(2)-O(12)	91.06(8)
N(3)-Mn(2)-O(8)	96.42(8)	N(3)-Mn(2)-O(1)ii	94.17(8)	N(3)-Mn(2)-O(12)	96.68(8)
O(5)iii-Mn(2)-O(8)	89.65(7)	O(1)ii-Mn(2)-O(5)iii	83.71(7)	O(5)iii-Mn(2)-O(12)	162.67(7)
O(5)iii-Mn(2)-N(3)	100.17(8)				

[a]Symmetry transformations used to generate equivalent atoms: i *x*, –*y*+3/2, *z*–1/2; ii –*x*+1, –*y*+1, –*z*+1for **1**; i –*x*, –*y*, –*z*; ii *x*, *y*–1, *z* for **2**; i –*x*+1, *y*, –*z*+1/2; ii –*x*+1, –*y*+2, –*z*+1; iii *x*–1, *y*, *z* for **3**; i –*x*+1, *y*, –*z*+3/2; ii –*x*+1, –*y*, –*z*+1; iii *x*+1, *y*, *z* for **4**; i –*x*, –*y*+1, –*z*+1; ii *x*–1, *y*, *z*; iii –*x*+1, –*y*, –*z*+1for **5**; i *x*–1/2, –*y*+3/2, *z*+1/2; ii –*x*+3/2, *y*+1/2, –*z*+3/2 for **6**; i –*x*+1, –*y*+1, –*z*+1; ii –*x*, –*y*, –*z*+2; iii –*x*, –*y*+1, –*z*+2 for **7**; i *x*, *y*+ 1, *z*; ii –*x*, –*y*+1, –*z*+2; iii –*x*+1, –*y*+1, –*z*+1 for **8**.

Table S2. Hydrogen Bonds in Crystal Packing [Å, °] of **1–8**.

Complexes	D-H…A	*d*(D-H)	*d*(H…A)	*d*(D…A)	∠DHA	Symmetry code
1	N(2)-H(2)···O(12)	0.762	2.222	2.952	160.99	*x*-1, *y*, *z*
	O(3)-H(5)···O(6)	0.860	1.711	2.531	158.54	-*x*+1, *y*+1/2, -*z*+3/2
	O(8)-H(4)···O(2)	0.820	1.673	2.477	166.04	-*x*, *y*-1/2, -*z*+1/2
	O(10)-H(1W)···O(12)	0.751	2.246	2.848	137.99	-*x*+2, *y*+1/2, -*z*+1/2
	O(10)-H(2W)···O(8)	0.853	2.149	2.994	170.06	-*x*+1, *y*+1/2, -*z*+1/2
	O(11)-H(3W)···O(6)	0.850	1.977	2.827	179.13	-*x*, -*y*+1, -*z*+1
	O(11)-H(4W)···O(12)	0.761	1.999	2.706	154.71	*x*-1, *y*, *z*
	O(12)-H(5W)···O(5)	0.850	2.057	2.870	159.83	-*x*+1, -*y*+1, -*z*+1

**2**	N(1)-H(1)···O(3)	0.860	2.102	2.902	154.64	-*x*+1, -*y*+1, -*z*+1
	N(2)-H(2)···O(3)	0.860	2.005	2.826	159.33	-*x*+1, -*y*+1, -*z*+1
	O(10)-H(1W)···O(6)	0.851	1.834	2.631	155.08	*x*+1, *y*, *z*+1
	O(10)-H(2W)···O(9)	0.851	2.153	2.789	131.25	-*x*+1, -*y*+1, -*z*+1
	O(11)-H(3W)···O(4)	0.850	2.063	2.912	178.90	
	O(11)-H(4W)···O(2)	0.850	1.915	2.765	179.51	-*x*+1, -*y*, -*z*+1
**3**	N(1)-H(1)···O(8)	0.860	2.495	3.247	152.55	-*x*+1, *y*, -*z*+3/2
	N(2)-H(2)···O(8)	0.860	2.246	3.079	163.00	-*x*+1, *y*, -*z*+3/2
	O(10)-H(1W)···O(12)	0.859	2.327	2.772	112.58	
	O(11)-H(3W)···O(4)	0.853	2.188	2.955	149.64	-*x*+1, -*y*+2, -*z*+1
	O(11)-H(4W)···O(7)	0.853	1.964	2.773	157.71	-*x*+1, -*y*+2, -*z*+1
**4**	N(1)-H(1)···O(7)	0.860	2.306	3.107	155.09	-*x*+1, *y*, -*z*+1/2
	N(2)-H(2)···O(7)	0.860	2.284	3.097	157.57	-*x*+1, *y*, -*z*+1/2
	O(10)-H(2W)···O(12)	0.906	1.871	2.757	165.41	
	O(11)-H(3W)···O(3)	0.923	2.041	2.917	157.97	-*x*+1, -*y*, -*z*+1
	O(11)-H(4W)···O(8)	0.917	1.927	2.786	155.14	-*x*+2, -*y*, -*z*+1
	O(12)-H(5W)···O(6)	0.850	1.924	2.735	159.13	-*x*+1, -*y*, -*z*+1
	O(12)-H(6W)···O(9)	0.850	2.372	2.785	110.41	-*x*+1, *y*, -*z*+3/2
**5**	N(4)-H(4)···O(1)	0.860	1.883	2.701	158.33	-*x*, -*y*+1, -*z*
	N(5)-H(5)···O(1)	0.860	2.134	2.906	149.18	-*x*, -*y*+1, -*z*
	N(8)-H(9)···O(7)	0.860	1.945	2.784	164.76	-*x*+2, -*y*, -*z*+1
	N(9)-H(10)···O(6)	0.860	1.817	2.676	177.99	-*x*+2, -*y*, -*z*+1
	N(12)-H(12)···O(4)	0.860	1.998	2.790	152.68	*x*-1, *y*, *z*
	N(13)-H(13)···O(4)	0.860	2.437	3.158	141.88	*x*-1, *y*, *z*
**6**	N(1)-H(1)···O(4)	0.900	1.960	2.854	171.86	-*x*+1/2, *y*+1/2, -*z*+3/2
	N(2)-H(2)···O(3)	0.885	1.875	2.750	169.60	-*x*+1/2, *y*+1/2, -*z*+3/2
	N(3)-H(4)···O(6)	0.860	1.848	2.699	177.03	*x*+1/2, -*y*+3/2, *z*+1/2
	N(4)-H(5)···O(14)	0.860	2.114	2.973	177.45	
	N(5)-H(7)···O(9)	0.860	1.854	2.687	162.67	-*x*+2, -*y*+1, -*z*+1
	O(10)-H(1W)···O(12)	0.850	1.945	2.771	163.76	-*x*+1, -*y*+1, -*z*+2
	O(10)-H(2W)···O(2)	0.850	2.086	2.747	134.16	
	O(11)-H(3W)···O(2)	0.850	1.890	2.717	163.79	-*x*+1, -*y*+1, -*z*+2
	O(11)-H(4W)···O(6)	0.850	2.033	2.797	149.15	*x*+1/2, -*y*+3/2, *z*+1/2
	O(12)-H(5W)···O(3)	0.872	2.030	2.844	154.86	*x*+1, *y*, *z*
	O(12)-H(6W)···O(14)	0.913	2.048	2.945	167.08	
	O(13)-H(7W)···O(8)	0.866	2.133	2.912	149.40	-*x*+3/2, *y*+1/2, -*z*+3/2
	O(13)-H(8W)···O(4)	0.865	2.120	2.774	131.93	-*x*+1/2, *y*+1/2, -*z*+3/2
	O(14)-H(9W)···O(13)	0.850	1.955	2.778	162.73	-*x*+3/2, *y*-1/2, -*z*+3/2
	O(14)-H(10W)···O(5)	0.850	1.901	2.746	172.38	
**7**	N(1)-H(1)···O(3)	0.860	2.240	3.040	154.71	-*x*+1, -*y*, -*z*+1
	N(2)-H(2)···O(3)	0.860	2.150	2.968	158.85	-*x*+1, -*y*, -*z*+1
	O(10)-H(1W)···O(5)	0.887	2.015	2.716	135.14	-*x*, -*y*+1, -*z*+2
	O(11)-H(3W)···O(6)	0.854	1.902	2.628	142.06	-*x*, -*y*, -*z*+2
	O(12)-H(5W)···O(7)	0.868	2.104	2.709	126.14	*x*, *y*+1, *z*
	O(12)-H(6W)···O(4)	0.867	1.991	2.835	163.88	-*x*+1, -*y*+1, -*z*+1

	O(13)-H(7W)···O(11)	0.852	2.017	2.859	169.31	
	O(13)-H(8W)···O(6)	0.852	1.882	2.719	167.13	*x*, *y*+1, *z*-1
**8**	N(1)-H(1)···O(5)	0.860	2.179	3.001	159.70	-*x*+1, -*y*, -*z*+1
	N(2)-H(2)···O(5)	0.860	2.269	3.065	154.00	-*x*+1, -*y*, -*z*+1
	O(9)-H(1W)···O(3)	0.851	1.935	2.659	142.23	*x*, *y*+1, *z*
	O(9)-H(2W)···O(14)	0.851	1.850	2.698	174.95	-*x*+1, -*y*+1, -*z*+2
	O(10)-H(3W)···O(15)	0.858	2.076	2.716	130.85	
	O(10)-H(4W)···O(13)	0.857	2.341	2.848	118.13	
	O(11)-H(5W)···O(6)	0.850	1.949	2.789	169.63	*x*-1, *y*, *z*+1
	O(11)-H(6W)···O(4)	0.850	1.907	2.706	155.99	-*x*, -*y*, -*z*+2
	O(12)-H(7W)···O(9)	0.852	2.014	2.832	160.77	-*x*, -*y*+1, -*z*+2
	O(12)-H(8W)···O(3)	0.851	1.896	2.734	167.83	*x*, *y*+1, *z*-1
	O(13)-H(9W)···O(15)	0.850	2.272	2.965	138.80	
	O(13)-H(10W)···O(6)	0.850	2.000	2.838	168.51	-*x*+1, -*y*+1, -*z*+1
	O(14)-H(11W)···O(6)	0.850	1.930	2.765	166.70	-*x*+1, -*y*+1, -*z*+1

Table S3. Comparison of Various Catalysts in the Coupling Reactions of Aldehydes with Nitromethane.

Entry	Catalyst	Catalyst (mol%)	Solvent	Time (h)	Temp. (°C)	Conversion (%)	Ref.
1	$[Cd_2(\mu_4\text{-cada})(H_2biim)_3]_n\cdot 2nH_2O$	4.0	MeOH	12	70	99	This work
2	$[Co_2(\mu_6\text{-cada})(\mu\text{-dpey})_{0.5}(H_2O)_4]_n\cdot 2nH_2O$	4.0	MeOH	12	70	93	This work
3	$[Mn_2(\mu_6\text{-cada})(\mu\text{-dpea})_{0.5}(H_2O)_4]_n\cdot 2nH_2O$	4.0	MeOH	12	70	91	This work
4	$Cu(p\text{-FBA})_2(MIm)_2$	1.0	CH_2Cl_2	24	25	85	48
5	Cu@HATN-CTF	3.0	EtOH	12	70	82	49
6	CuL_1	5.0	EtOH	12	25	90	50
7	CuL_2	5.0	H_2O	2	25	78	50

p-FBA: *p*-fluorobenzoic acid; MIm: 1–methylimidazole; HATN: 5,6,11,12,17,18-hexaazatrinaphthylene; CTF: Covalent Triazine Framework; L_1: *N*,*N*-dibenzylcyclohexane-1,2-diamine; L_2: *N*,*N*-bis(pyridin-2-ylmethyl)cyclohexane-1,2-diamine.
